# Life’s essential 8 and specific cancer risk and mortality in men and women: a population-based cohort analysis of 332,417 United Kingdom participants

**DOI:** 10.1186/s12885-025-14048-5

**Published:** 2025-04-08

**Authors:** Chuang Yang, Wenke Cheng, Patrick S. Plum, Florian Lordick, Jeanette Köppe, Ines Gockel, René Thieme

**Affiliations:** 1 Department of Visceral, Transplant, Thoracic and Vascular Surgery, University Hospital Leipzig, Leipzig, Germany; 2 Medical Faculty, University of Leipzig, Leipzig, Germany; 3 Department of Oncology, Gastroenterology, Hepatology and Pulmonology, University Hospital Leipzig, Leipzig, Germany; 4 Institute of Biostatistics and Clinical Research, University of Muenster, Muenster, Germany

**Keywords:** Life’s essential 8, Cardiovascular health (CVH), Cancer occurrence, Mortality, UK biobank

## Abstract

**Background:**

This study aimed to explore the association between Life’s Essential 8 (LE8) and the risk of cancer occurrence and cancer-associated mortality across 24 cancer types. The cardiovascular health (CVH) score is constructed based on the overall LE8 score, providing a more direct measure of CVH and its potential relationship with cancer risk.

**Methods:**

This cohort enrolled participants from a prospective cohort of the United Kingdom Biobank, including individuals aged 37–73 years, with 332,417 cancer-free participants. CVH scores were assessed using the LE8 metrics. The primary outcome of this study was the risk of cancer events, and the secondary outcome was cancer mortality. Competitive models were used to examine the associations between each 10-point increment in the CVH score and the outcomes, with stratified analyses conducted for both men and women to assess sex differences.

**Results:**

The mean CVH score was 64.4(55.6,72.5) in men and 70.0 (61.2,78.1) in women (*P* < 0.001). During a mean follow-up time of 12.0 years, 12.32% (95% confidence interval [CI]: 12.21–12.43%) of participants developed cancer, and 2.13% (95% CI: 2.08–2.18%) died from cancer. A 10-point rise in CVH score was negatively associated with overall cancer occurrence in men (hazard ratio [HR]: 0.97, 95% CI: 0.96–0.98) and women (HR: 0.96, 95% CI: 0.95–0.97), along with reduced cancer mortality risk in both sexes. Moreover, sex differences were observed in the impact of a 10-point CVH increase on esophageal, gastric, colorectal, and liver cancers.

**Conclusions:**

Lower CVH scores were associated with an increased overall cancer risk and higher cancer-related mortality, highlighting the need for cancer screening in patients with low CVH scores.

**Supplementary Information:**

The online version contains supplementary material available at https://doi.org/10.1186/s12885-025-14048-5.

## Introduction

As the global population ages, the burdens of cancer incidence and mortality increase rapidly [1]. Cancer ranks as the primary or secondary cause of death in over a hundred countries, posing a significant threat to human life [2]. However, up to half of cancer cases can be averted by reducing exposure to well-known risk factors such as tobacco and alcohol consumption, physical inactivity, an unhealthy diet, and obesity [3].

The American Heart Association (AHA) introduced a cardiovascular health (CVH) score to prevent cardiovascular diseases (CVD) in 2010, comprising seven lifestyle factors called Life’s Simple 7: smoking status, body weight, total cholesterol, blood glucose, blood pressure, physical activity, and diet [4]. Previous studies have established a clear link between Life’s Simple 7 (LS7) and various CVDs, such as coronary heart disease, cerebral small vessel disease, and atrial fibrillation [4–6], with higher LS7 scores (indicating better CVH) being associated with a lower risk of these conditions. In 2022, the AHA expanded the CVH framework by introducing Life’s Essential 8 (LE8), an updated cardiovascular health metric that incorporates sleep behavior and refines the scoring algorithm [7]. LE8 consists of eight independent components, each with its own individual score, and the overall LE8 score is used to construct the updated CVH score. Sleeping behavior has a crucial role in metabolism, immune function, and inflammation, all of which can be linked to cancer development [8–10]. Insufficient sleep leads to oxidative stress, immune dysfunction, hormonal imbalances, and chronic inflammation, potentially increasing cancer risk [11]. Additionally, sleep interacts with other lifestyle factors, such as physical activity, diet, and obesity, further influencing overall health [12–14]. Despite growing evidence on sleep’s role in chronic diseases, its association with cancer incidence and mortality has not been fully explored. As an enhancement of LS7, LE8 provides a more comprehensive and precise assessment of CVH, yet its potential relevance to cancer risk remains largely unexamined.

Numerous studies have demonstrated that higher levels of CVH based on LE8 are associated with a reduced risk of CVD and other conditions like non-alcoholic fatty liver, diabetes, and even all-cause mortality [15–19]. Cancer, a severe life-threatening disease, is influenced by various lifestyle factors, with significant sex differences in occurrence, incidence, and mortality rates [1, 20, 21]. Many epidemiological studies examining the link between unhealthy lifestyles (smoking, alcohol consumption, and obesity) and cancer risk have not conducted separate analyses based on sex [22–24]. Lin et al. examined the association between CVH score and cancer mortality in the US NHANES and UK Biobank cohorts [25]. However, their study primarily focused on overall cancer mortality and no exploration on the associations between CVH and specific cancer entities or potential sex differences were done.

In a prospective cohort study of 502,357 participants from the United Kingdom (UK), we aimed to comprehensively analyze the sex-specific effects of LE8 on the incidence and mortality of 24 different types of cancer and 10 histologic cancer subtypes. We hypothesize that higher LE8 scores, reflecting better CVH, are associated with a lower risk of both cancer occurrence and cancer-related mortality. Given the substantial socioeconomic burden of cardiovascular disease and cancer. This study aims to explore the potential role of an integrated CVH metric in cancer prevention and prognosis.

## Methods

### Study participants

**Fig. 1:**
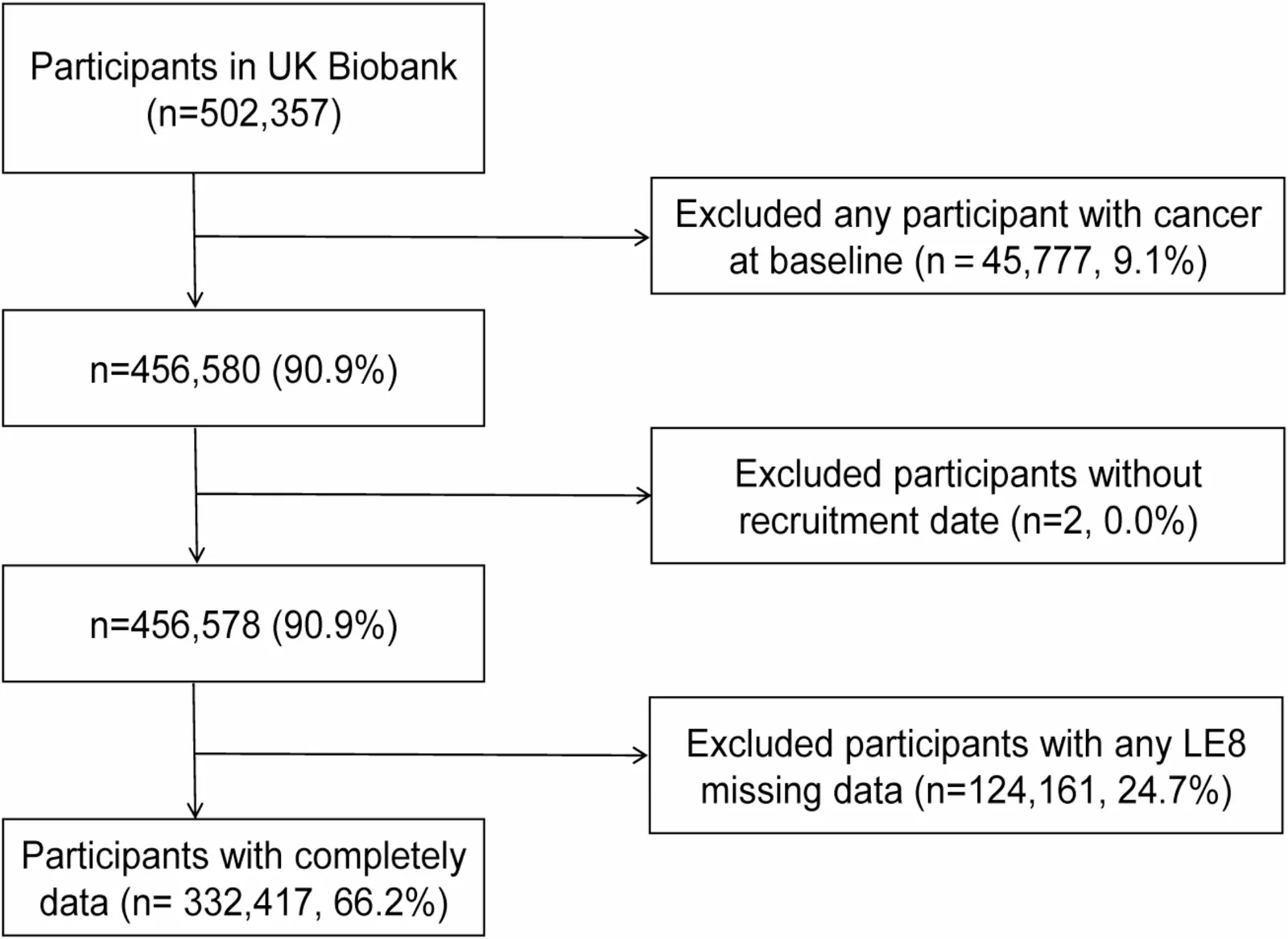
Flow diagram illustrating the derivation of participant selection. LE8, Life’s Essential 8

The UK Biobank (UKB) conducted a prospective cohort study from 2006 to 2010, enrolling 502,357 participants aged 37–73 years from diverse UK regions. This extensive study explored genetic, lifestyle, and environmental factors contributing to various diseases [26]. The project described in this study was approved by the UKB (107335). For our study, participants already diagnosed with cancer at recruitment (*n* = 45,777), two without recruitment date, and missing LE8 data (*n* = 124,161) were excluded, resulting in a study cohort of 332,417 participants with complete LE8 data (Fig. 1).

### Assessment of CVH and LE8 metrics

The CVH score was extracted from the LE8, encompassing eight health behaviors and factors: diet, blood pressure, blood glucose, sleep health, body mass index (BMI), physical activity, tobacco/nicotine exposure, and blood lipids [7]. Each LE8 metric was assigned a score ranging from 0 to 100 and the CVH score was calculated as the unweighted mean of the eight component scores, meaning that each metric contributed equally to the final score. Following AHA guidelines, CVH scores were categorized into three levels: Low CVH: CVH score < 50; Moderate CVH: CVH score 50 ≤ CVH < 80; High CVH: CVH score ≥ 80 [7]. Further details on the scoring criteria for each metric were provided in Tables S1 and S2.

### Covariates

Sociodemographic variables included, were age, sex (male, female), ethnicity (white, other ethnicities), education level, Townsend deprivation index (TDI), annual household income (<£18,000, £18,000–29,999, £30,000–51,999, £52,000–100,000, and >£100,000), alcohol status (never, previous, current, and prefer not to answer), menstrual status, and CVD status. Education levels were categorized into five levels from one to five, according to the International Standard Classification of Education Codes (ISCED, Table S3) [27]. For participants who chose “Prefer not to answer” or had missing data, we considered those values as unknown. The missing data are completely missing at random, and the details were shown in Table S4.

### Outcome ascertainment

The primary outcomes of this study encompassed both total and individual cancer risks across all entities, identified using the International Classification of Diseases, 10th Revision (ICD-10) codes (Table S5) [28]. Additionally, we investigated 10 histological cancer subtypes (Table S6). Cancers types, lacking clear histological subtypes were categorized as “other types” without detailed investigation.

The secondary outcome of this study encompassed the risk of mortality attributable to various causes. The censoring date was defined as the last occurrence date of disease (June 1, 2022) and death (December 19, 2022) data. The observation period for participants commenced at enrollment and continued until the first occurrence of loss to follow-up, cancer diagnosis, or death. For individual cancer analyses, we focused on cancers with at least 100-recorded events by the end of follow-up.

### Statistical analysis

In the descriptive analysis, Chi-square test was used to compare differences between categorical variables, and the Mann-Whitney U test was used to compare differences between continuous variables. To assess the correlation among LE8 metrics, Spearman correlation coefficients were computed separately for male and female phenotypic data. This was done using the “corrplot” function in R, and the results were visualized through correlation heatmaps [29]. Statistical comparisons of CVH differences between the two groups were conducted using the “ggpubr” package and assessed with a Mann-Whitney-U test [30].

In the primary outcomes analysis, considering death as an independently occurring competing event, we used a competitive risk model by the “mstate” package [31]. For the secondary endpoint, at the last follow-up date, participants were initially categorized into death and non-death at the last follow-up, analyzed using multivariable Cox regression. The proportional hazards assumption was tested using Schoenfeld residuals, and no significant violations were detected. Subsequently, to investigate cancer mortality, deaths were classified into cancer, non-cancer-related, and unknown deaths (not further studied). Finally, we analyzed the data on deaths caused by cancer, focusing on those caused by various specific cancers. Within each set of death data, we categorized deaths into specific cancer deaths, other cancer deaths, non-cancer deaths, and deaths of unknown causes. Competitive models were applied to study each target mortality type. Models were fully adjusted for age, ethnicity, education level, annual household income, TDI, alcohol status, baseline CVD status, and menopausal status for women. Co-variable selection in the final model was adjusted based on previous studies [16, 18, 32].

These models were employed to estimate the sub-distributional hazard ratios (HRs) and their corresponding 95% confidence intervals (CIs) concerning the relationships between LE8 and CVH scores (for each 10-point increase or high, moderate, and low CVH groups) and the risk of cancer or mortality.

The comparison of cumulative incidence rates utilized the Fine-Gray test [33]. In the overall cancer study, we plotted cumulative incidence curves for observed and competitive events. However, in the individual cancer study, for clarity, only cumulative incidence curves for observed events were plotted.

To further investigate sex differences in the relationship between every 10-point increase in CVH and cancer occurrence risk, subgroup analyses were conducted (age ethnicity, CVD, TDI, education, annual household income, menopausal status, and alcohol status).

To evaluate the robustness of the association between CVH and cancer occurrence, two sensitivity analyses were performed, which consisted of two parts. Individuals diagnosed with cancer within the initial two years of follow-up were excluded to mitigate reverse causality, and multiple imputation methods were used to impute missing variables, resulting in five complete datasets. Furthermore, we compared the results with outcomes obtained through mean imputation.

Balloon plots were used to visually assess the effect of increasing LE8 by each 10-point increment on cancer occurrence. The size of each balloon indicated the HR value, whereas the color reflected the correlation (positive or negative). Statistical significance was set at *P* < 0.05, with significance markers *, **, and *** representing *P* < 0.05, *P* < 0.01, and *P* < 0.001, respectively. The study was considered fully explorative, and all results were interpreted accordingly.

All statistical analyses were performed using R (http://www.R-project.org, version 4.3.1).

## Results

**Fig. 2:**
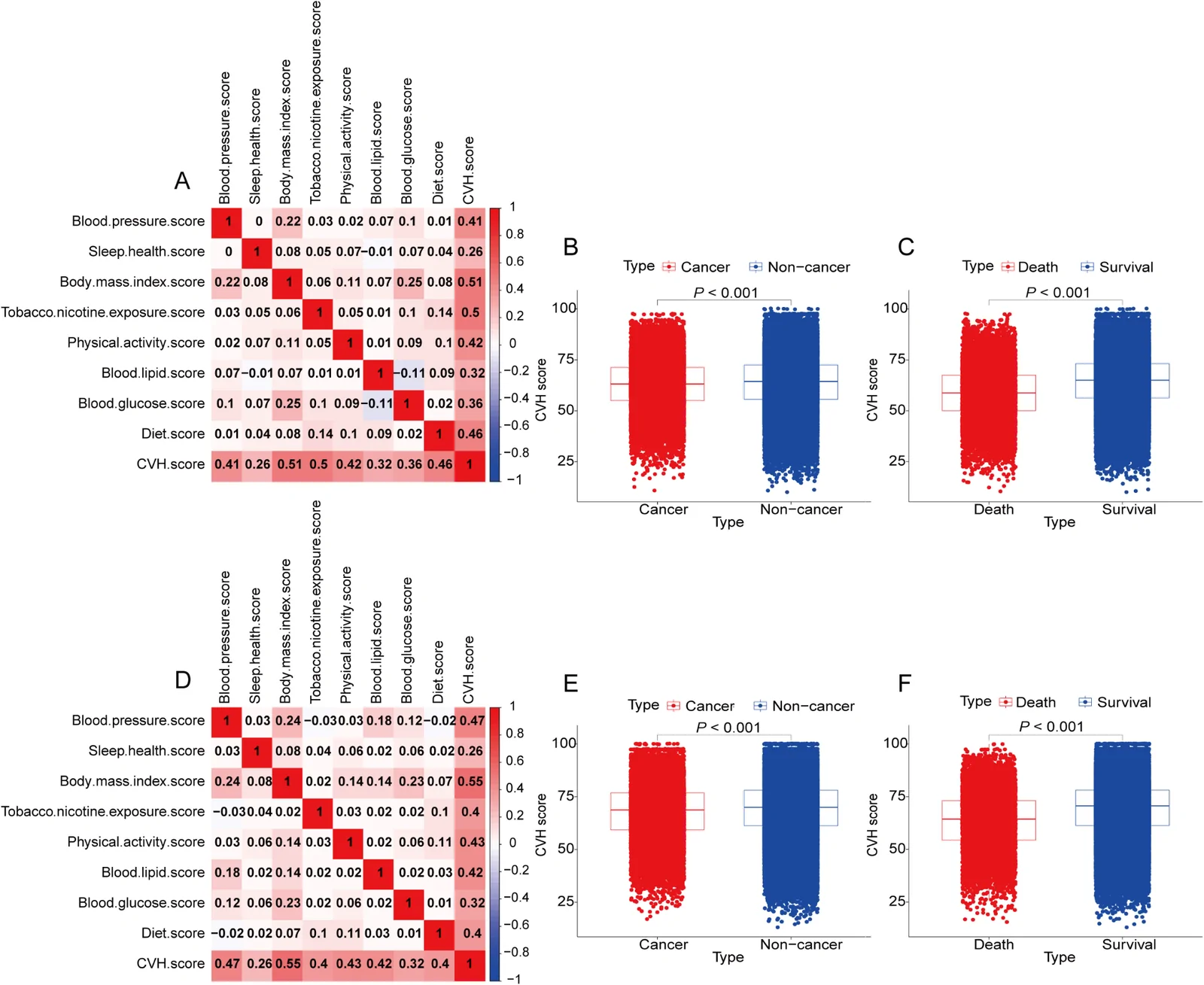
Correlation analysis between LE8 components and variations in CVH scores in the population. (**A**) Heat map of correlations between LE8 metrics in men. (**B**) Differences in CVH scores between male participants with and without cancer. (**C**) Differences in CVH scores between alive and deceased male participants. (**D**) Heat map of correlations between LE8 metrics in women. (**E**) Differences in CVH scores between female participants with and without cancer. (**F**) Differences in CVH scores between alive and deceased female participants. LE8, Life’s Essential 8; CVH, cardiovascular health

Of the 502,357 recorded participants, 332,417 cancer-free participants were included in this study (Table 1). Over the 10-year follow-up period after enrollment, 12.32% (95% CI: 12.21–12.43%) of the participants developed cancer. Among men, various components of the LE8 score showed a weak correlation, whereas a strong correlation was observed with the CVH score. The highest correlation was observed between CVH and BMI scores (Fig. 2A). Differential analysis revealed notably higher baseline total CVH scores in male participants who remained cancer-free or survived compared to those who developed cancer or died (Fig. 2B and C). Similar trends were observed in female participants (Fig. 2D and F).

**Table 1:** Baseline characteristics and incident cancer cases during follow-up included men and women

Characteristic	Men (*n* = 158,334)	Women (*n* = 174,083)	*P*-value
Baseline characteristics N (%)			
**Age (years)**	58 (50, 63)	57 (49, 63)	< 0.001
**Ethnicity**			0.194
White	150,447 (95.0%)	165,240 (94.9%)	
Others	7887 (5.0%)	8,843 (5.1%)	
**Education levels**			< 0.001
1	24,405 (15.4%)	26,408 (15.2%)	
2	38,303 (24.2%)	50,241 (28.9%)	
3	16,963 (10.7%)	21,472 (12.3%)	
4	7,013 (4.4%)	9,939 (5.7%)	
5	70,396 (44.5%)	64,663 (37.1%)	
Unknown	1,254 (0.8%)	1,360 (0.8%)	
**Annual household income before tax**,** £**			< 0.001
< 18,000	27,118 (17.1%)	33,904 (19.5%)	
18,000–30,999	34,061 (21.5%)	38,120 (21.9%)	
31 000–51,999	39,389 (24.9%)	38,442 (22.1%)	
52,000-100,000	33,870 (21.4%)	29,126 (16.7%)	
> 100,000	9,422 (6.0%)	7,684 (4.4%)	
Unknown /Not prefer to answer	14,474 (9.1%)	26,807 (15.4%)	
**Townsend deprivation index**	-2.2 (-3.7,0.4)	-2.2 (-3.7,0.3)	0.417
**Alcohol status**			< 0.001
Never	3,935 (2.5%)	9,403 (5.4%)	
Previous	5,303 (3.3%)	5,997 (3.4%)	
Current	148,997 (94.1%)	158,585 (91.1%)	
Prefer not to answer	99 (0.1%)	98 (0.1%)	
**Menopause**	NA		
Yes		103,382 (59.4%)	
No		44,308 (25.5%)	
Unknown		26,393 (15.2%)	
**CVD**	16,273 (10.3%)	8,625 (5.0%)	< 0.001
**AHA Life’s Essential 8 score**			
Total CVH score	64.4 (55.6,72.5)	70 (61.2, 78.1)	< 0.001
Diet score	50 (25, 80)	50 (25, 80)	< 0.001
Blood pressure score	25 (5, 50)	50 (25, 75)	< 0.001
Blood glucose score	100 (100, 100)	100 (100, 100)	< 0.001
Sleep health score	100 (70, 100)	100 (70, 100)	< 0.001
Body mass index score	70 (30, 70)	70 (70, 100)	< 0.001
Physical activity score	100 (100, 100)	100 (90, 100)	< 0.001
Tobacco/nicotine exposure score	50 (25, 100)	75 (25, 100)	< 0.001
Blood lipid score	40 (20, 60)	40 (20, 60)	< 0.001
**Cancer types and subtypes [Incident cases**,** (%)]**			
**Overall cancer**	27,706 (17.5%)	23,121 (13.3%)	< 0.001
**Anus**	49 (0.0%)	77 (0.0%)	0.049
Bladder	513 (0.3%)	170 (0.1%)	< 0.001
Brain	297 (0.2%)	216 (0.1%)	< 0.001
Breast	44 (0.0%)	6,114 (3.5%)	< 0.001
Colorectal	2,212 (1.4%)	1,644 (0.9%)	< 0.001
Esophageal	469 (0.3%)	175 (0.1%)	< 0.001
Esophageal adenocarcinoma (EAC)	351 (0.2%)	66 (0.0%)	< 0.001
Esophageal squamous cell carcinoma (ESCC)	74 (0.0%)	93 (0.1%)	0.39
Kidney	614 (0.4%)	313 (0.2%)	< 0.001
Renal cell carcinoma (RCC)	529 (0.3%)	272 (0.2%)	< 0.001
Transitional cell carcinoma (TCC)	40 (0.0%)	19 (0.0%)	0.002
Laryngeal	115 (0.1%)	5 (0.0%)	< 0.001
Leukemia	495 (0.3%)	315 (0.2%)	< 0.001
Liver	289 (0.2%)	130 (0.1%)	< 0.001
Hepatocellular carcinoma (HCC)	176 (0.1%)	27 (0.0%)	< 0.001
Cholangiocarcinoma (CAC)	92 (0.1%)	95 (0.1%)	0.668
Lung	1,350 (0.9%)	1,205 (0.7%)	< 0.001
Small cell carcinoma (SCLC)	163 (0.1%)	176 (0.1%)	0.868
Non-small cell carcinoma (NSCLC)	982 (0.6%)	894 (0.5%)	0.325
Lymphoma	826 (0.5%)	677 (0.4%)	< 0.001
Hodgkin’s lymphoma (HL)	52 (0.0%)	42 (0.0%)	0.136
Non-Hodgkin’s lymphoma (NHL)	743 (0.5%)	602 (0.3%)	< 0.001
Mesothelioma	208 (0.1%)	52 (0.0%)	< 0.001
Multiple Myeloma	352 (0.2%)	245 (0.1%)	< 0.001
Oral	435 (0.3%)	223 (0.1%)	< 0.001
Ovarian	NA	637 (0.4%)	
Pancreatic	462 (0.3%)	381 (0.2%)	< 0.001
Prostate	7,725 (4.9%)	NA	
Melanoma skin	10,018 (6.3%)	8,280 (4.8%)	< 0.001
Small intestine	87 (0.1%)	78 (0.0%)	0.19
Soft tissue	115 (0.1%)	168 (0.1%)	0.018
Stomach	319 (0.2%)	143 (0.1%)	< 0.001
Thyroid	81 (0.1%)	211 (0.1%)	< 0.001
Uterine	NA	1,003 (0.6%)	

CVD, cardiovascular disease, CVH, cardiovascular health

### Per 10-point increment in CVH and LE8 scores were associated with cancer occurrence and exhibited different effects in men and women

With each 10-point increase in the CVH score, the overall risk of cancer significantly decreased. This association remained consistent in both men (HR: 0.97, 95% CI: 0.96–0.98) and women (HR: 0.96, 95% CI: 0.95–0.97) after adjusting for all potential confounders (Table 2). Moreover, this negative association was also observed between CVH scores and different cancers in men and women (all *P* < 0.05, Table 2), showing that a lower CVH is associated with a higher risk of developing cancer.

**Table 2:** Association between per 10-point increment CVH score and the risk of cancer in male and female

Cancer Type	Male	Female	*P* for interaction
	HR (95%CI)	HR (95%CI)	
Overall	0.97 (0.96–0.98) ^c^	0.96 (0.95–0.97) ^c^	0.839
Oral	0.82 (0.76–0.88) ^c^	0.83 (0.75–0.93) ^c^	0.333
Esophageal	0.71 (0.66–0.77) c	0.89 (0.79–1.01)	0.001
EAC	0.69 (0.64–0.76) ^c^	0.78 (0.64–0.95) ^a^	0.228
ESCC	0.78 (0.65–0.94) ^a^	1.07 (0.9–1.27)	0.004
Stomach	0.79 (0.72–0.86) ^c^	0.93 (0.82–1.07)	0.044
Small intestine	0.86 (0.72–1.03)	1.03 (0.85–1.24)	0.284
Colorectal	0.86 (0.83–0.89) ^c^	0.94 (0.9–0.97) ^b^	0.002
Anus	0.95 (0.75–1.2)	1.01 (0.83–1.21)	0.458
Liver	0.64 (0.58–0.7) ^c^	0.83 (0.72–0.95) ^b^	< 0.001
HCC	0.57 (0.51–0.64) ^c^	0.78 (0.57–1.05)	0.02
CAC	0.8 (0.68–0.95) ^a^	0.85 (0.72-1) ^a^	0.575
Pancreatic	0.84 (0.78–0.91) ^c^	0.84 (0.77–0.91) ^c^	0.891
Laryngeal	0.66 (0.56–0.76) ^c^	1.09 (0.51–2.32)	0.134
Lung	0.67 (0.64–0.7) ^c^	0.7 (0.67–0.73) ^c^	0.185
SCLC	0.65 (0.57–0.73) ^c^	0.65 (0.58–0.73) ^c^	0.87
NSCLC	0.68 (0.65–0.72) ^c^	0.72 (0.68–0.76) ^c^	0.062
Melanoma skin	1.08 (1.06–1.1) ^c^	1.09 (1.07–1.11) ^c^	0.686
Mesothelioma	1.03 (0.91–1.15)	0.97 (0.77–1.22)	0.916
Soft tissue	0.99 (0.85–1.16)	0.94 (0.83–1.07)	0.815
Breast	0.91 (0.71–1.17)	0.94 (0.92–0.96) ^c^	0.526
Uterine	NA	0.8 (0.76–0.84) ^c^	
Ovarian	NA	0.99 (0.93–1.06)	
Prostate	1.05 (1.03–1.07) ^c^	NA	
Kidney	0.82 (0.77–0.87) ^c^	0.79 (0.72–0.86) ^c^	0.517
RCC	0.84 (0.78–0.9) ^c^	0.79 (0.72–0.87) ^c^	0.292
TCC	0.77 (0.59-1) ^a^	0.72 (0.5–1.04)	0.936
Bladder	0.79 (0.73–0.85) ^c^	0.91 (0.8–1.03)	0.246
Brain	0.95 (0.87–1.05)	1.04 (0.93–1.17)	0.183
Thyroid	0.93 (0.78–1.12)	0.98 (0.88–1.1)	0.529
Lymphoma	1.01 (0.95–1.07)	1.03 (0.97–1.1)	0.681
HL	0.9 (0.71–1.13)	0.91 (0.71–1.16)	0.97
NHL	1.03 (0.96–1.09)	1.07 (1-1.15) ^a^	0.42
Multiple myeloma	0.99 (0.91–1.09)	0.96 (0.86–1.06)	0.421
Leukemia	0.96 (0.89–1.03)	0.99 (0.9–1.08)	0.524

CVH, cardiovascular disease health; Models were fully adjusted with age, ethnicity, education level, annual household income, Townsend deprivation index, alcohol status, and baseline CVD status. Additionally, for female, the model was further adjusted menopausal status. a: *P* < 0.05, b: *P* < 0.01, c: *P* < 0.001

However, higher CVH scores appeared to be a favorable factor for men with melanoma skin and prostate cancer as well as women with melanoma skin and NHL (Table 2). Additionally, a 10-point increase in CVH was associated with sex differences in esophageal, colorectal, and liver cancer occurrence (*P* for interaction < 0.05).

**Fig. 3:**
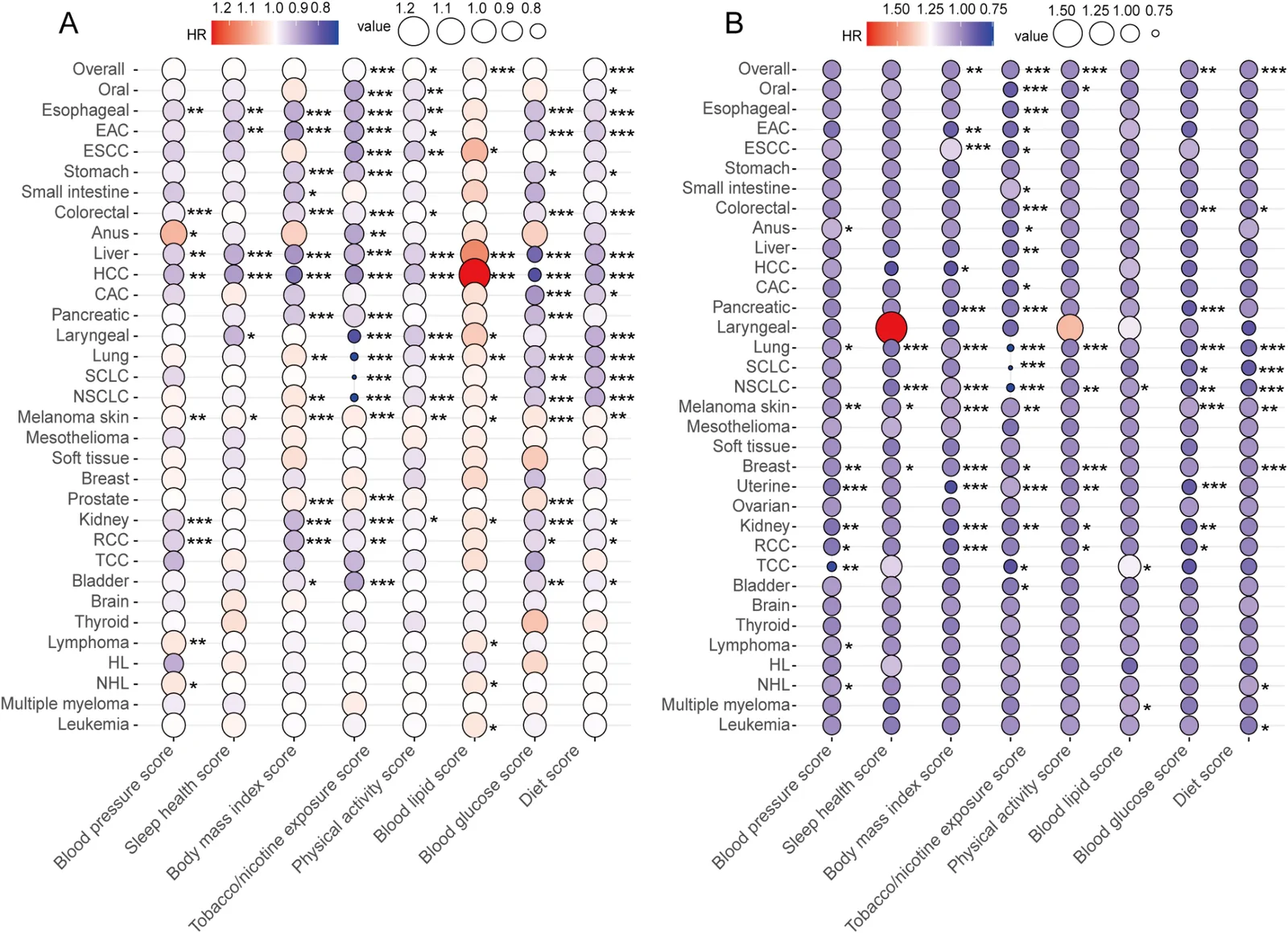
Balloon plots of LE8 metrics and any site-specific cancer. The association between LE8 metrics and different cancers in men (**A**) and women (**B**). *P < 0.05, **P < 0.01, ***P < 0.001. The size of each balloon indicated the HR value, and the color reflected the correlation. Blue color represented positive correlation, and red color represented negative correlation. LE8, Life’s Essential 8; HR, hazard ratio; CVH, cardiovascular health

Subsequently, the association between each 10-point increment in the individual LE8 metrics and cancer risk was assessed. Most cancer entities were associated with at least one LE8 metric score in both men and women. Among these eight metrics, tobacco/nicotine exposure and BMI scores showed the strongest associations with cancer occurrence in men and women, respectively (Fig. 3A and B, Tables S6 and S7). However, a 10-point increase in the blood lipid score was associated with various cancers in men (overall, ESCC, liver, HCC, laryngeal, lung, NSCLC, skin melanoma, kidney, lymphoma, NHL, and leukemia) and women (NSCLC, TCC, and multiple myeloma) (Fig. 3, Tables S7 and S8).

### Association between CVH levels and cancer risk

Subsequently, participants were divided into low, moderate, and high CVH groups. Compared to the low CVH score group, participants in the moderate and high CVH score groups showed significantly lower overall cancer occurrence in men (HR: 0.89, 95% CI: 0.84–0.94; Table 3) and women (HR: 0.85, 95% CI: 0.81–0.9; Table 3). However, male and female participants with high CVH had an increased risk of developing melanoma skin cancer by 35% and 33%, respectively, compared to those with low and moderate CVH (Table 3).

**Table 3:** Association between CVH levels and the risk of cancer occurrence in male and female

Cancer site	Male			Female		
	Ref	HR (95% CI)	HR (95% CI)	Ref	HR (95% CI)	HR (95% CI)
	Low	Moderate	High	Low	Moderate	High
Overall	1	0.95 (0.91–0.98) ^b^	0.89 (0.84–0.94) ^c^	1	0.88 (0.84–0.93) ^c^	0.85 (0.81–0.9) ^c^
Oral	1	0.7 (0.55–0.9) ^b^	0.48 (0.3–0.76) ^b^	1	0.55 (0.37–0.82) ^b^	0.45 (0.27–0.74) ^b^
Esophageal	1	0.61 (0.49–0.77) ^c^	0.14 (0.07–0.3) ^c^	1	0.58 (0.37–0.9) ^a^	0.5 (0.28–0.9) ^a^
EAC	1	0.56 (0.44–0.72) ^c^	0.07 (0.02–0.21) ^c^	1	0.47 (0.24–0.92) ^a^	0.28 (0.1–0.77) ^a^
ESCC	1	0.88 (0.48–1.6)	0.5 (0.14–1.76)	1	1.01 (0.48–2.11)	1.04 (0.43–2.51)
Stomach	1	0.71 (0.54–0.94) ^a^	0.24 (0.12–0.51) ^c^	1	0.87 (0.5–1.5)	0.69 (0.34–1.4)
Small intestine	1	0.89 (0.5–1.59)	0.52 (0.17–1.59)	1	1.16 (0.49–2.72)	1.75 (0.67–4.59)
Colorectal	1	0.77 (0.69–0.86) ^c^	0.56 (0.46–0.69) ^c^	1	0.89 (0.74–1.06)	0.81 (0.66-1) ^a^
Anus	1	0.93 (0.43–2.02)	0.71 (0.18–2.76)	1	1.57 (0.57–4.37)	1.17 (0.37–3.72)
Liver	1	0.42 (0.32–0.54) ^c^	0.13 (0.06–0.3) ^c^	1	1.03 (0.55–1.94)	0.87 (0.4–1.87)
HCC	1	0.36 (0.26–0.5) ^c^	0.07 (0.02–0.28) ^c^	1	1.19 (0.27–5.16)	1.03 (0.18–5.86)
CAC	1	0.54 (0.33–0.88) ^a^	0.23 (0.07–0.77) ^a^	1	1.02 (0.49–2.14)	0.77 (0.31–1.93)
Pancreatic	1	0.71 (0.56–0.9) ^b^	0.44 (0.27–0.71) ^c^	1	0.57 (0.43–0.77) ^c^	0.4 (0.27–0.61) ^c^
Laryngeal	1	0.43 (0.29–0.64) ^c^	0.13 (0.03–0.57) ^b^		NA	NA
Lung	1	0.51 (0.45–0.58) ^c^	0.16 (0.1–0.24) ^c^	1	0.49 (0.42–0.56) ^c^	0.24 (0.19–0.31) ^c^
SCLC	1	0.46 (0.33–0.65) ^c^	0 (0-4.68)	1	0.44 (0.3–0.64) ^c^	0.33 (0.18–0.59) ^b^
NSCLC	1	0.53 (0.46–0.62) ^c^	0.18 (0.11–0.28) ^c^	1	0.54 (0.45–0.64) ^c^	0.24 (0.18–0.32) ^c^
Melanoma skin	1	1.2 (1.13–1.28) ^c^	1.35 (1.24–1.48) ^c^	1	1.12 (1.03–1.23) ^a^	1.33 (1.2–1.46) ^c^
Mesothelioma	1	0.99 (0.68–1.45)	1.03 (0.55–1.95)	1	0.73 (0.31–1.76)	0.81 (0.27–2.42)
Soft tissue	1	0.73 (0.44–1.2)	1.06 (0.52–2.17)	1	0.86 (0.49–1.5)	0.93 (0.49–1.78)
Breast	1	0.89 (0.39–2.03)	0.5 (0.1–2.45)	1	0.91 (0.83-1)	0.8 (0.72–0.9) ^c^
Uterine		NA	NA	1	0.62 (0.51–0.76) ^c^	0.43 (0.33–0.55) ^c^
Ovarian		NA	NA	1	1.16 (0.85–1.59)	1.06 (0.74–1.52)
Prostate	1	1.15 (1.08–1.24) ^c^	1.11 (1-1.23)		NA	NA
Kidney	1	0.7 (0.57–0.85) ^c^	0.47 (0.32–0.7) ^c^	1	0.67 (0.48–0.95) ^a^	0.34 (0.21–0.56) ^c^
RCC	1	0.76 (0.6–0.95) ^a^	0.52 (0.34–0.8) ^b^	1	0.67 (0.47–0.97) ^a^	0.37 (0.22–0.62) ^c^
TCC	1	0.66 (0.3–1.46)	0.74 (0.19–2.83)	1	0.28 (0.1–0.82) ^a^	0.1 (0.01–0.86) ^a^
Bladder	1	0.64 (0.51–0.79) ^c^	0.42 (0.27–0.66) ^c^	1	0.8 (0.49–1.29)	0.8 (0.43–1.47)
Brain	1	0.85 (0.62–1.17)	0.9 (0.55–1.47)	1	1.15 (0.66-2)	1.37 (0.74–2.53)
Thyroid	1	0.78 (0.42–1.44)	0.86 (0.35–2.13)	1	0.99 (0.59–1.67)	0.88 (0.48–1.6)
Lymphoma	1	1.03 (0.84–1.26)	1.04 (0.76–1.41)	1	1.01 (0.76–1.35)	1.18 (0.85–1.64)
HL	1	0.7 (0.34–1.47)	0.71 (0.21–2.33)	1	0.38 (0.16–0.9) ^a^	0.63 (0.23–1.72)
NHL	1	1.05 (0.85–1.31)	1.12 (0.81–1.54)	1	1.13 (0.82–1.55)	1.34 (0.93–1.92)
Multiple myeloma	1	1 (0.73–1.36)	0.99 (0.62–1.59)	1	1.02 (0.64–1.63)	0.87 (0.5–1.53)
Leukemia	1	0.83 (0.65–1.06)	0.8 (0.54–1.19)	1	1.29 (0.82–2.02)	0.98 (0.58–1.67)

CVH, cardiovascular disease health; Ref, reference; Models were fully adjusted with age, ethnicity, education level, annual household income, Townsend deprivation index, alcohol status, and baseline CVD status. Additionally, for female, the model was further adjusted menopausal status. a: *P* < 0.05, b: *P* < 0.01, c: *P* < 0.001

Furthermore, the cumulative incidence functions revealed notable differences in cancer risk between men (Fig. S1) and women (Fig. S2) with different CVH scores. The pattern indicated that increasing CVH grade corresponded to a gradual decrease in the cumulative cancer incidence rate. However, a low CVH score correlated with a higher cumulative incidence of melanoma skin cancer in both sexes and prostate cancer in men (Figs. S1 and S2).

### CVH and cancer mortality

Within 10 years after enrollment, 5.53% of men and 2.89% of women participants died, with 2.59% of men and 1.72% of women dying due to cancer (Table S9).

A 10-point increase in the CVH score correlated with a notable reduction in all-cause mortality, deaths from non-cancer causes, and deaths attributed to cancer (Table S8). Additionally, we observed sex differences in the effect of each 10-point increase in the CVH score on all-cause mortality, non-cancer, overall cancer, small intestine, and liver cancer mortality (all *P* for interaction < 0.05, Table S8).

The low CVH group, compared to the moderate and high CVH score groups, showed a reduced risk of all-cause mortality, deaths from non-cancer causes, and cancer-related deaths in men and women (Table S10).

The cumulative incidence function (event rates) of cancer mortality analysis indicated a reduction in the risk of cancer mortality with an increase in CVH score in men (Fig. S3) and women (Fig. S4).

### Subgroup analysis

Stratified analyses based on potential risk factors revealed that every 10-point increase in the CVH score notably reduced the risk of cancer, with interactions between the TDI and history of alcohol consumption (Table S11). Interactions were observed in both men and women within the CVD and alcohol consumption groups, and further interaction was noted among men for household income (Table S11).

### Sensitivity analysis

The results remained consistent with the initial analysis when we excluded participants with cancer events occurring within two years from the baseline during the follow-up period (Fig. S5). Additionally, a high degree of consistency was found between the results of multiple imputations and mean imputation (Table S12 and Fig. S6).

## Discussion

In this large-scale population-based prospective cohort study, we represented the first comprehensive assessment of the association between CVH, defined by LE8 metrics, and cancer occurrence and mortality risk. CVDs and malignant diseases impose a high socioeconomic burden on society and the health system. Putative associations between these health issues may result in more effective prevention programs, especially for high-risk groups. CVDs are linked to higher overall mortality, leading to lower quality of life and altered functional outcomes [34]. Healthcare professionals must recognize that CVH and associated CVDs can increase the risk of cancer occurrence and cancer-related mortality. Any primary physicians, such as general practitioner should consider early cancer detection and recommend screening programs, especially for patients with low CVH levels, to prevent cancer occurrence in this population.

We found that the LE8 component scores, except for the blood lipid score, were negatively associated with the risk of different cancer entities. Male and female participants with lower baseline total CVH scores had higher gender-specific risks of cancer and cancer-related mortality than those with higher CVH scores.

Previous studies have only investigated the associations between individual risk factors, such as body weight, diet, smoking, and alcohol consumption, and cancer risk [35–38]. Our study results indicate that each component of the LE8 score was associated with the occurrence of at least one cancer entity in both male and female participants. After combining all eight subcomponents, the overall CVH score exhibited a stronger association with cancer risk.

LS7, developed by the AHA, has been extensively studied and is well-established in assessing CVDs risk, further validating its effectiveness as a widely used metric for CVH evaluation [4–6]. Additionally, Van et al. investigated the relationship between CVH, as measured by LS7, and cancer risk in a cohort of 13,933 participants. Their findings demonstrated that each 1-point increase in LS7 score was associated with a reduced overall cancer incidence, and specific cancer risk including breast, lung, prostate, and colorectal cancers [15]. Moreover, women with the lowest LS7 scores had a significantly higher cancer risk compared to those with the highest scores, further emphasizing the importance of maintaining an optimal CVH [31]. Among all cancer types, ideal LS7 scores exhibited the strongest inverse association with lung cancer risk [31]. Furthermore, higher LS7 scores were linked to a reduced overall cancer incidence and mortality, underscoring LS7’s clinical utility in risk stratification and preventive strategies.

To further refine CVH assessment, LE8 was introduced as an enhanced version of LS7, incorporating sleep health as an additional metric, acknowledging the growing evidence of poor sleeping behavior to both CVDs and cancer [8–10]. A recent study conducted by Herraiz-Adillo Á et al. reported that LE8 outperforms LS7 in assessing CVH, providing a more comprehensive evaluation [39].

By integrating LE8 into a large-scale cohort study (> 500,000 participants), our study expands upon previous LS7-based findings, while providing a more comprehensive and nuanced framework for CVH. This will not only allow validating prior LS7 findings, but also offers new insights into the association between an enhanced CVH model and cancer incidence and mortality.

Currently, knowledge of the correlation between CVH assessment and cancer-related mortality is limited. Recently, in a small cohort study (*n* = 127), no correlation was observed between the baseline CVH status and cancer patient mortality [40]. Gao et al. found that individuals with ideal CVH scores had a 62% lower risk of cancer-related mortality than those with low CVH scores [41]. In a recent study of participants with chronic kidney disease, each 10-point increase in LE8 score correlated with a 12% reduction in cancer-related mortality. However, no association was observed between different levels of CVH and cancer-related mortality, affirming our findings that CVH impacts cancer occurrence and mortality [42]. Additionally, in large-scale studies, Zhao Y et al. found that better CVH serves as a protective factor for both breast cancer incidence and mortality [43]. Similarly was reported by Lin L et al., which investigated the association between LE8 and pan-cancer mortality risk, finding that optimal CVH contributes to a lower risk of cancer-related mortality [25]. Compared to these studies, our study not only examined the relationship between overall LE8 scores and pan-cancer incidence and mortality but also evaluated the impact of each individual LE8 component. Furthermore, we conducted sex-stratified analyses to minimize potential bias arising from gender differences. Our study highlights the sex heterogeneity in the association between LE8 and cancer risk, particularly in digestive system cancers. Several digestive system malignancies, including colorectal, liver, and pancreatic cancers, exhibited notable sex disparities in their association with CVH levels. Previous studies have suggested that biological differences, including hormonal regulation, metabolic processes, and immune responses, may contribute to these sex-based variations in cancer risk. For instance, estrogen is thought to have a protective effect against colorectal and liver cancers in women, while higher visceral fat deposition and metabolic syndrome prevalence in men may contribute to increased digestive cancer risk [44–46]. Additionally, differences in lifestyle factors, such as alcohol consumption, smoking, and dietary patterns between men and women, may further influence the observed sex disparity in LE8-related cancer risk [47].

A large-scale study on blood pressure and various cancer entities indicated a positive association between systolic blood pressure (SBP) and cancer occurrence. However, a weak inverse relationship exists between SBP and cervical SCC or lymphoma [48]. Comparing men with normal SBP (< 130 mmHg) and those with SBP > 150 mmHg (aged > 45 years), a 35% increased risk of developing prostate cancer was shown. Furthermore, among patients with prostate cancer, those with SBP > 150 mmHg had a 49% increased overall mortality rate compared to patients with normal SBP [49]. Our findings also indicate that blood pressure scores are associated with the occurrence of cancer.

Elevated blood sugar levels are positively associated with cancer risk and mortality, aligning with our results [50–52]. Additionally, we found that an increase in lipid scores appeared to increase cancer risk in both men and women, suggesting that decreased non-HDL-C levels may contribute to increased cancer risk. Currently, an ongoing debate exists within the academic community regarding this claim [53–56].

The AHA introduced sleep health scores into the CVH score in 2022, as a strong correlation between poor sleep health and the risk breast, lung, esophageal, and stomach cancers has been shown [16]. This was consistent with our findings, especially in men [14, 57, 58].

CVH score was closely associated with the occurrence or mortality of most cancers in both men and women, primarily showing a negative correlation. Interestingly, a high CVH score appears to be associated with an increased risk of skin melanoma and prostate cancer; however, the underlying mechanism remains unknown.

However, the potential limitations of this study must be considered. First, the LE8 metrics relied primarily on self-reported questionnaires from the UK Biobank, which may introduce information bias [59, 60]. This could lead to misclassification of certain CVH components, potentially attenuating the observed associations between CVH and cancer risk. Second, approximately 25% of participants with missing LE8 values were excluded, which may introduce selection bias. If the excluded individuals tended to have poorer CVH, the true association between CVH and cancer incidence or mortality could be underestimated. Third, information on most CVH metrics was only available at baseline, without consideration of dynamic changes over life span. This limitation could lead to an underestimation of the long-term effects of CVH fluctuations on cancer risk and mortality. Fourth, as most participants in the UK Biobank cohort were Caucasians, the generalizability of our findings to other ethnicities may be limited. If similar associations hold in more diverse populations, the observed effect sizes might differ. Finally, despite adjusting for confounding factors, participants’ medication history was not fully accounted for. Given the observational nature of the study, certain medications (e.g., lipid-lowering drugs or antihypertensives) may have influenced both CVH and cancer risk, potentially biasing the results toward the null.

## Conclusions

Maintaining a healthy lifestyle and achieving a high LE8 score may significantly reduce the risk of cancer risk and mortality. Higher LE8 scores were associated with an approximately 11% and 62% lower risk of cancer incidence and mortality in men, and a 15% and 59% reduction in women, respectively. Given the strong link between low CVH levels and increased risk of both CVD and cancer, improving CVH through modifiable lifestyle factors is crucial for reducing the overall disease burden and improving long-term health outcomes. Public health efforts should emphasize early intervention and preventive strategies targeting individuals with low LE8 scores, aiming to mitigate the dual risk of CVD and cancer.

## Electronic supplementary material

Below is the link to the electronic supplementary material.

Supplementary Material 1

## Data Availability

The datasets analyzed during the current study are available in a public, open access repository. The UK Biobank data are available for approved researchers through the UK Biobank data-access protocol.
