# Supplementary material for: Life’s essential 8 and specific cancer risk and mortality in men and women: a population-based cohort analysis of 332,417 United Kingdom participants
Source: BMC Cancer. 2025 Apr 8;25:632. doi: 10.1186/s12885-025-14048-5 (PMC11980174; doi:10.1186/s12885-025-14048-5)
Supplement: Supplementary file 1 — Supplementary Material 1 [file 12885_2025_14048_MOESM1_ESM.docx]

**Life’s Essential 8 and Specific Cancer Risk and Mortality in Men and Women: A Population-based Cohort Analysis of 332,417 United Kingdom Participants**

**Chuang Yang, Wenke Cheng, Patrick S. Plum, Florian Lordick, Jeanette Köppe, Ines Gockel, René Thieme**

[Table S1. Scoring criteria of diet in UK Biobank according to the Diet score 4](#_Toc190900556)

[Table S2. Assessment Methods for Each Individual CVH Metric 5](#_Toc190900557)

[Table S3. UK Biobank self‐report highest qualification mapped to International Standard for Classification of Education codes 7](#_Toc190900558)

[Table S4. Information on missing covariates among 332,417 UK Biobank participants 8](#_Toc190900559)

[Table S5. ICD-10 codes for each cancer 9](#_Toc190900560)

[Table S6. Histological subtypes of cancer in UK Biobank 10](#_Toc190900561)

[Table S7. Hazard ratios (95% confidence intervals) of LE8 metrics per 10 points increment associated with individual cancer risk in men 11](#_Toc190900562)

[Table S8. Hazard ratios (95% confidence intervals) of LE8 metrics per 10 points increment associated with individual cancer risk in women 13](#_Toc190900563)

[Table S9. The association between per 10 points increment of CVH and the risk of cancer mortality in men and women 15](#_Toc190900564)

[Table S10. Associations between CVH levels and risk of cancer mortality in men and women 17](#_Toc190900565)

[Table S11. Subgroup analysis of the correlation between per 10-point increment in CVH score in clinical variables and cancer risk 18](#_Toc190900566)

[Table S12. The association between per 10 points increment of CVH and cancer risk with multiple imputations in men and women 20](#_Toc190900567)

[Fig. S1. Cumulative incidence of cancers according to CVH categories in men. 22](#_Toc190900568)

[24](#_Toc190900569)

[Fig. S2. Cumulative incidence of cancers according to CVH categories in women. 24](#_Toc190900570)

[Fig. S3. Cumulative incidence curve of cancer mortality according to CVH categories in men. 26](#_Toc190900571)

[Fig. S4. Cumulative incidence curve of cancer mortality according to CVH categories in women. 27](#_Toc190900572)

[Fig. S5. Sensitivity analysis of the association between per 10-point increment in CVH score and the risk of cancer after excluding cancer events occurring within two years from the baseline in men (A) and women (B). 28](#_Toc190900573)

[Fig. S6. Sensitivity analysis of the association between per 10-point increment CVH score and the risk of cancer with multiple imputation in men (A) and women (B). 29](#_Toc190900574)

[Reference 30](#_Toc190900575)

Table S1. Scoring criteria of diet in UK Biobank according to the Diet score

| **Diet component ^1^** | **Scoring Criteria** | **Score** |
| --- | --- | --- |
| Fruit & vegetables | <5 serving/day | 1: If scoring condition met  0: If scoring condition not met (Range: 0-9) |
| Total fish intake | < once a week of each one |  |
| Processed meat | > Once a week |  |
| Red meat | ≤Once a week |  |
| Milk type used | Full cream/ another type of milk/ never rarely have milk |  |
| Spread type | Another selection |  |
| Cereal intake | ≤5 bowls |  |
| Salt added to food | Sometimes/usually/always |  |
| Water intake | < 6glasses |  |

| Table S2. Assessment Methods for Each Individual CVH Metric | | | |
| --- | --- | --- | --- |
| CVH metric ^2^ | Points | Diet score | Metrics: Diet score |
| Diet score | 100 | 0-2 | The self-completed touch-screen question-naire (completed at baseline) was used to collect the frequency of consumption of food items during the previous year. 9 food items were dichotomized into meeting and  not meeting recommendations using cut-offs.  1-point was assigned to participants for each unhealthy category met^1^. |
|  | 80 | 3-4 |  |
|  | 50 | 5 |  |
|  | 25 | 6 |  |
|  | 0 | 7-9 |  |
| Physical activity score | Points | Physical activity | Metrics: minutes of moderate- intensity activity per week |
|  | 100 | ≥150 minutes | Participants reported their weekly minutes of moderate or vigorous physical activity. One minute of vigorous activity is deemed equivalent to two minutes of moderate activity. |
|  | 90 | 120-149 minutes |  |
|  | 80 | 90-119 minutes |  |
|  | 60 | 60-89 minutes |  |
|  | 40 | 30-59 minutes |  |
|  | 20 | 1-29 minutes |  |
|  | 0 | 0 minutes |  |
| Smoking score | Points | Smoking | Metrics: Tobacco/nicotine  /Secondhand smoke exposure |
|  | 100 | Never smoker | Participants self-reported their tobacco use, encompassing current smoking status and smoking history, as well as exposure to secondhand smoke. Participants living with an active indoor smoker at home had 20 points deducted from their score, unless their score was zero.  Participants who indicated they 'smoked on most or all days in the past.' were classified as “Former smoker, quit <1 years”.  Participants who indicated they 'smoked occasionally in the past' were classified as “Former smoker, quit 1-5 years”.  Participants who reported having 'just tried once or twice in the past' were classified as “Former smoker, quit ≥ 5 years”. |
|  | 75 | Former smoker, quit ≥ 5years |  |
|  | 50 | Former smoker, quit 1-5years |  |
|  | 25 | Former smoker, quit < 1 years |  |
|  | 0 | Current smoker |  |
| Sleep health score | Points | Sleep duration | Metrics: Sleep hours |
|  | 100 | 7-9 hours | Participants reported their average nightly sleep duration. |
|  | 90 | 9-10 hours |  |
|  | 70 | 6-7 hours |  |
|  | 40 | 5-6 or ≥10 hours |  |
|  | 20 | 4–5 hours |  |
|  | 0 | < 4 hours |  |
| Body mass index score | Points | Body mass index | Metrics: Body mass index |
|  | 100 | <25 kg/m^2^ | Height was determined using the Seca 202 height measuring instrument. Weight was precisely measured to the nearest 0.1 kg using the Tanita BC-418 MA body composition analyzer. Body mass index (BMI) was calculated as weight (in kg) divided by the square of height (in meters). |
|  | 70 | 25.0-30.0 kg/m^2^ |  |
|  | 30 | 30.0-35 kg/m^2^ |  |
|  | 15 | 35.0-40 kg/m^2^ |  |
|  | 0 | ≥ 40 kg/m^2^ |  |
| Blood lipid score | Point | Non-HDL cholesterol | Metrics: Non-HDL cholesterol |
|  | 100 | < 130 mg/dL | Non-HDL cholesterol was derived by subtracting HDL cholesterol from total cholesterol. Serum cholesterol levels were determined enzymatically.  If treated with medication, deduct 20 points. |
|  | 60 | 130-159 mg/dL |  |
|  | 40 | 160-189 mg/dL |  |
|  | 20 | 190-219 mg/dL |  |
|  | 0 | 220 mg/dL |  |
| Blood glucose score | Points | HbA1c | Metrics: HbA1c |
|  | 100 | No diabetes and HbA1c<5.7% | HbA1c levels were determined using high-performance liquid chromatography techniques. |
|  | 60 | No diabetes and HbA1c 5.7–6.4% |  |
|  | 50 | No diabetes and HbA1c >6.4% |  |
|  | 40 | Diabetes with HbA1c<7.0% |  |
|  | 30 | Diabetes with HbA1c 7.0-8.0% |  |
|  | 20 | Diabetes with HbA1c 8.0-9.0% |  |
|  | 10 | Diabetes with HbA1c 9.0-10.0% |  |
|  | 0 | Diabetes with HbA1c≥10.0 |  |
| Blood pressure score | Points | Blood pressure | Metrics: Systolic and diastolic BPs |
|  | 100 | <120 / <80 mmHg | The mean of all available blood pressure readings was utilized to determine both systolic and diastolic blood pressures.  Subtract 20 points from the score (unless the score is 0) if under treatment. |
|  | 75 | 120-130 / < 80 mmHg |  |
|  | 50 | 130-140 or 80-90 mmHg |  |
|  | 25 | 140-160 or 90-100 mmHg |  |
|  | 0 | ≥ 160 or ≥ 100 mmHg |  |

| Table S3. UK Biobank self‐report highest qualification mapped to International Standard for Classification of Education codes | | |
| --- | --- | --- |
|  |  |  |
| **Qualification (As reported in UK Biobank)** | **ISCED** | **Estimated years of education** |
| College or University degree | 5 | 20 |
| NVQ or HND or HNC or equivalent | 5 | 19 |
| Other prof. qual. eg: nursing, teaching | 4 | 15 |
| A levels/AS levels or equivalent | 3 | 13 |
| O levels/GCSEs or equivalent | 2 | 10 |
| CSEs or equivalent | 2 | 10 |
| None of the above | 1 | 7 |

| Table S4. Information on missing covariates among 332,417 UK Biobank participants | | |  |
| --- | --- | --- | --- |
| **Covariates** | **Number of missing** | **Missing rates (%)** | ***P*** |
| Race and ethnicity | 870 | 0.26 | 0.857 |
| Townsend Deprivation Index | 405 | 0.12 | 0.690 |
| Education levels | 2,614 | 0.79 | 0.999 |
| Annual household income before tax | 41281 | 12.42 | 1.000 |
| Alcohol status | 197 | 0.06 | 0.595 |
| Menopause | 26,393 | 7.94 | 1.000 |

Little’s MCAR Test was used to assess the nature of missing data. A *P*-value < 0.05 suggests that the missing data follow a missing at random (MAR) pattern, whereas a *P*-value > 0.05 indicates that the missing data are missing completely at random (MCAR).

| Table S5. ICD-10 codes for each cancer | |
| --- | --- |
| **Cancer Type** | **ICD-10 codes** |
| Overall | C00-C97 |
| Oral | C00-14 |
| Esophageal | C15 |
| Stomach | C16 |
| Small intestine | C17 |
| Colorectal | C18, C19, C20 |
| Anus | C21 |
| Liver | C22 |
| Pancreatic | C25 |
| Laryngeal | C32 |
| Lung | C33, C34 |
| Melanoma skin | C43,44 |
| Mesothelioma | C45 |
| Soft tissue | C46, C47, C48, C49 |
| Breast | C50 |
| Uterine | C54, C55 |
| Ovarian | C56 |
| Prostate | C61 |
| Kidney | C64, C65 |
| Bladder | C67 |
| Brain | C71 |
| Thyroid | C73 |
| Lymphoma | C81, C82, C83, C84, C85, C86, C88 |
| Multiple myeloma | C90 |
| Leukemia | C91, C92, C93, C94, C95 |

| Table S6. Histological subtypes of cancer in UK Biobank | | |
| --- | --- | --- |
| **Cancer Type** | **Histological subtype** | **UK Biobank data field 40011 values** |
| Esophageal cancer | Adenocarcinoma (EAC) | 8140, 8144, 8210, 8260, 8323, 8480, 8211, 8574, 8481 |
|  | Squamous cell carcinoma (ESCC) | 8070, 8071 |
|  | Other types | 8000, 8010, 8020, 8145, 8246, 8490, 8560, 8012, 8041, 8720, 8990, 8045 |
| Kidney cancer | Renal cell carcinoma (RCC) | 8031, 8032, 8050, 8140, 8260, 8270, 8290, 8310, 8312, 8317, 8318, 8323, 8480, 8890, 8959, 8964 |
|  | Transitional cell carcinoma (TCC) | 8120, 8130, 8122 |
|  | Other types | 8000, 8010, 8858, 8070, 8960, 9590 |
| Liver cancer | Hepatocellular carcinoma (HCC) | 8170, 8033 |
|  | Cholangiocarcinoma (CAC) | 8140, 8160. 8162, 8180 |
|  | Other types | 8000, 8010, 8480, 9120, 9133, 8890, 8241, 9491, 8240, 8246, 9071, 8800 |
| Lung cancer | Small cell carcinoma (SCLC) | 8041, 8045, 8246, 8240, 8042 |
|  | Non-small cell carcinoma (NSCLC) | 8012, 8013, 8020, 8022, 8032, 8033, 8046, 8050, 8070, 8071, 8072, 8074, 8140, 8200, 8230, 8244, 8250, 8251, 8252, 8253, 8255, 8260, 8310, 8323, 8430, 8440, 8480, 8481, 8490, 8550, 8560, 8574, 8800, 8972, 8980, 9040, 9120 |
|  | Other types | 8000, 8010, 8011, 8021, 8249, 8802, 8810, 9591, 9590 |
| Lymphoma | Hodgkin’s lymphoma (HL) | 9650, 9652, 9659, 9663, 9665, 9651, 9664, 9667, 9653, 9661 |
|  | Non-Hodgkin’s lymphoma (NHL) | 9591, 9670, 9671, 9673, 9675, 9680, 9684, 9687, 9689, 9690, 9691, 9695, 9698, 9699, 9700, 9701, 9702, 9705, 9709, 9714, 9715, 9717, 9718, 9719, 9724, 9727, 9761 |
|  | Other types | 8000, 9590, 9760 |

| Table S7. Hazard ratios (95% confidence intervals) of LE8 metrics per 10 points increment associated with individual cancer risk in men | | | | | | | | |
| --- | --- | --- | --- | --- | --- | --- | --- | --- |
| **Cancer Type** | **Blood pressure score** | **Sleep health score** | **Body mass index score** | **Tobacco/nicotine exposure score** | **Physical activity score** | **Blood lipid score** | **Blood glucose score** | **Diet score** |
| Overall | 1 (0.99-1) | 1 (0.99-1) | 1 (1-1) | 0.99 (0.98-0.99) ^c^ | 1 (0.99-1) ^a^ | 1.01 (1.01-1.01) ^c^ | 1 (0.99-1) | 0.99 (0.99-0.99) ^c^ |
| Oral | 0.98 (0.94-1.01) | 0.97 (0.93-1.02) | 1.03 (1-1.07) | 0.89 (0.87-0.91) ^c^ | 0.96 (0.94-0.98) ^b^ | 0.99 (0.96-1.03) | 1.02 (0.97-1.07) | 0.97 (0.94-1) ^a^ |
| Esophageal | 0.95 (0.92-0.99) ^b^ | 0.94 (0.9-0.98) ^b^ | 0.9 (0.87-0.93) ^c^ | 0.9 (0.88-0.93) ^c^ | 0.96 (0.94-0.98) ^b^ | 1.03 (1-1.06) | 0.92 (0.89-0.96) ^c^ | 0.95 (0.92-0.97) ^c^ |
| EAC | 0.96 (0.92-1) | 0.92 (0.88-0.97) ^b^ | 0.88 (0.85-0.92) ^c^ | 0.89 (0.87-0.92) ^c^ | 0.97 (0.94-1) ^a^ | 1.02 (0.99-1.06) | 0.92 (0.88-0.96) ^c^ | 0.93 (0.9-0.96) ^c^ |
| ESCC | 0.94 (0.86-1.02) | 0.94 (0.84-1.04) | 1.03 (0.95-1.13) | 0.88 (0.82-0.94) ^c^ | 0.93 (0.88-0.98) ^b^ | 1.1 (1.01-1.19) ^a^ | 1 (0.9-1.1) | 0.96 (0.89-1.03) |
| Stomach | 0.97 (0.93-1.01) | 0.98 (0.92-1.03) | 0.93 (0.89-0.97) ^c^ | 0.92 (0.89-0.95) ^c^ | 0.99 (0.96-1.02) | 1.02 (0.98-1.06) | 0.93 (0.88-0.97) ^a^ | 0.96 (0.92-0.99) ^a^ |
| Small intestine | 0.93 (0.86-1.01) | 0.96 (0.86-1.08) | 0.92 (0.86-1) ^a^ | 1.01 (0.96-1.08) | 0.97 (0.92-1.02) | 1.06 (0.98-1.14) | 0.9 (0.83-0.98) | 0.99 (0.92-1.05) |
| Colorectal | 0.97 (0.95-0.98) ^c^ | 1 (0.97-1.02) | 0.95 (0.93-0.96) ^c^ | 0.97 (0.96-0.98) ^c^ | 0.99 (0.97-1) ^a^ | 1 (0.98-1.01) | 0.96 (0.94-0.97) ^c^ | 0.97 (0.96-0.99) ^c^ |
| Anus | 1.1 (1.01-1.21) ^a^ | 0.97 (0.84-1.12) | 1.06 (0.95-1.18) | 0.89 (0.82-0.97) ^b^ | 0.98 (0.91-1.06) | 1.04 (0.95-1.15) | 1.06 (0.92-1.23) | 0.94 (0.86-1.03) |
| Liver | 0.94 (0.9-0.98) ^b^ | 0.9 (0.85-0.94) ^c^ | 0.87 (0.83-0.9) ^c^ | 0.91 (0.88-0.94) ^c^ | 0.94 (0.91-0.97) ^c^ | 1.16 (1.11-1.21) ^c^ | 0.81 (0.78-0.85) ^c^ | 0.9 (0.86-0.93) ^c^ |
| HCC | 0.91 (0.86-0.97) ^b^ | 0.88 (0.82-0.93) ^c^ | 0.83 (0.79-0.88) ^c^ | 0.86 (0.82-0.9) ^c^ | 0.92 (0.89-0.95) ^c^ | 1.28 (1.21-1.36) ^c^ | 0.77 (0.73-0.81) ^c^ | 0.89 (0.84-0.93) ^c^ |
| CAC | 0.95 (0.88-1.03) | 1.02 (0.91-1.14) | 0.93 (0.86-1) | 0.98 (0.93-1.04) | 0.98 (0.93-1.04) | 1.04 (0.96-1.11) | 0.87 (0.8-0.94) ^c^ | 0.93 (0.87-0.99) ^a^ |
| Pancreatic | 0.99 (0.95-1.02) | 0.97 (0.92-1.02) | 0.93 (0.9-0.96) ^c^ | 0.95 (0.93-0.98) ^c^ | 0.99 (0.97-1.02) | 1.03 (1-1.06) | 0.91 (0.88-0.95) ^c^ | 0.98 (0.95-1.01) |
| Laryngeal | 0.99 (0.92-1.06) | 0.91 (0.84-0.99) ^a^ | 1 (0.93-1.07) | 0.77 (0.72-0.83) ^c^ | 0.93 (0.89-0.97) ^c^ | 1.07 (1-1.14) ^a^ | 0.97 (0.89-1.04) | 0.9 (0.85-0.96) ^c^ |
| Lung | 1.01 (0.99-1.03) | 0.98 (0.96-1.01) | 1.03 (1.01-1.05) ^b^ | 0.72 (0.71-0.74) ^c^ | 0.96 (0.95-0.97) ^c^ | 1.03 (1.01-1.05) ^b^ | 0.93 (0.91-0.95) ^c^ | 0.9 (0.88-0.91) ^c^ |
| SCLC | 0.95 (0.9-1.01) | 1 (0.92-1.08) | 1 (0.94-1.05) | 0.71 (0.66-0.76) ^c^ | 0.98 (0.94-1.02) | 1.01 (0.95-1.06) | 0.92 (0.87-0.98) ^b^ | 0.91 (0.86-0.96) ^c^ |
| NSCLC | 1.01 (0.99-1.03) | 0.98 (0.95-1.02) | 1.03 (1.01-1.06) ^b^ | 0.72 (0.7-0.74) ^c^ | 0.96 (0.95-0.98) ^c^ | 1.02 (1-1.05) ^a^ | 0.93 (0.91-0.95) ^c^ | 0.9 (0.88-0.92) ^c^ |
| Melanoma skin | 1.01 (1-1.02) ^b^ | 1.01 (1-1.03) ^a^ | 1.02 (1.01-1.02) ^c^ | 1.02 (1.01-1.02) ^c^ | 1.01 (1-1.01) ^b^ | 1.01 (1-1.01) ^a^ | 1.03 (1.02-1.04) ^c^ | 1.01 (1-1.02) ^b^ |
| Mesothelioma | 0.96 (0.91-1.02) | 0.96 (0.89-1.02) | 1.02 (0.97-1.08) | 1 (0.96-1.04) | 1.02 (0.98-1.06) | 1.02 (0.97-1.07) | 1.01 (0.95-1.08) | 1.01 (0.97-1.06) |
| Soft tissue | 1.01 (0.95-1.08) | 0.96 (0.87-1.05) | 1.04 (0.97-1.12) | 0.99 (0.94-1.04) | 0.96 (0.91-1) | 1.03 (0.96-1.09) | 1.07 (0.97-1.18) | 1 (0.94-1.06) |
| Breast | 1.01 (0.96-1.18) | 0.98 (0.84-1.15) | 0.96 (0.86-1.07) | 1.02 (0.94-1.11) | 0.95 (0.88-1.02) | 1.05 (0.94-1.16) | 0.92 (0.81-1.04) | 0.95 (0.86-1.05) |
| Prostate | 1 (0.99-1.01) | 1.01 (0.99-1.02) | 1.02 (1.01-1.03) ^c^ | 1.02 (1.01-1.02) ^c^ | 1.01 (1-1.01) | 1 (0.99-1) | 1.04 (1.02-1.05) ^c^ | 1 (0.99-1) |
| Kidney | 0.95 (0.92-0.98) ^c^ | 0.99 (0.94-1.03) | 0.91 (0.88-0.93) ^c^ | 0.96 (0.94-0.98) ^c^ | 0.98 (0.96-1) ^a^ | 1.03 (1-1.06) ^a^ | 0.94 (0.91-0.98) ^c^ | 0.97 (0.95-1) ^a^ |
| RCC | 0.94 (0.91-0.98) ^c^ | 0.99 (0.95-1.04) | 0.91 (0.88-0.94) ^c^ | 0.97 (0.94-0.99) ^b^ | 0.99 (0.96-1.01) | 1.03 (1-1.06) | 0.95 (0.92-0.99) ^a^ | 0.97 (0.94-1) ^a^ |
| TCC | 0.91 (0.8-1.04) | 1.02 (0.86-1.22) | 0.92 (0.82-1.03) | 0.91 (0.83-1) | 0.97 (0.89-1.05) | 1.04 (0.93-1.17) | 0.89 (0.79-1.01) | 1.02 (0.92-1.12) |
| Bladder | 0.98 (0.95-1.02) | 0.97 (0.92-1.01) | 0.96 (0.93-1) ^a^ | 0.89 (0.87-0.91) ^c^ | 0.99 (0.96-1.01) | 0.99 (0.96-1.03) | 0.95 (0.91-0.98) ^b^ | 0.97 (0.94-1) ^a^ |
| Brain | 0.97 (0.92-1.01) | 1.03 (0.97-1.1) | 1.01 (0.97-1.06) | 1 (0.97-1.03) | 0.99 (0.96-1.03) | 0.98 (0.94-1.02) | 0.97 (0.92-1.02) | 0.99 (0.96-1.03) |
| Thyroid | 0.99 (0.91-1.07) | 1.04 (0.92-1.18) | 0.99 (0.91-1.07) | 0.97 (0.91-1.03) | 0.96 (0.91-1.01) | 0.98 (0.91-1.06) | 1.08 (0.95-1.22) | 1.02 (0.95-1.09) |
| Lymphoma | 1.03 (1.01-1.06) ^b^ | 0.99 (0.95-1.02) | 0.98 (0.95-1) | 1 (0.98-1.02) | 0.99 (0.97-1.01) | 1.03 (1.01-1.06) ^a^ | 0.98 (0.95-1.01) | 1 (0.98-1.02) |
| HL | 0.9 (0.8-1.01) | 1.02 (0.87-1.19) | 0.98 (0.89-1.09) | 0.99 (0.92-1.07) | 0.98 (0.91-1.06) | 0.97 (0.88-1.07) | 1.05 (0.91-1.21) | 1 (0.91-1.09) |
| NHL | 1.03 (1.01-1.06) ^a^ | 1 (0.96-1.04) | 0.98 (0.95-1.01) | 1 (0.98-1.02) | 1 (0.98-1.02) | 1.03 (1.01-1.06) ^a^ | 0.99 (0.96-1.03) | 1 (0.98-1.02) |
| Multiple myeloma | 0.97 (0.94-1.01) | 0.97 (0.91-1.02) | 1 (0.96-1.04) | 1.02 (0.99-1.05) | 1 (0.97-1.03) | 1 (0.96-1.04) | 1.01 (0.96-1.06) | 1 (0.97-1.04) |
| Leukemia | 1 (0.96-1.03) | 1.01 (0.96-1.06) | 0.99 (0.96-1.02) | 0.98 (0.95-1) | 0.99 (0.97-1.02) | 1.03 (1-1.07) ^a^ | 0.98 (0.94-1.02) | 0.99 (0.96-1.01) |

HRs are calculated per 10-point increment in the score. LE8, Life's Essential 8; EAC, esophageal adenocarcinoma; ESCC, esophageal squamous cell carcinoma; HCC, hepatocellular carcinoma; CAC, cholangiocarcinoma; SCLC, small cell carcinoma; NSCLC, non-small cell carcinoma; RCC, renal cell carcinoma; TCC, transitional cell carcinoma; HL, Hodgkin's lymphoma; NHL, non-Hodgkin's lymphoma.

Models were fully adjusted with age, ethnicity, education level, annual household income, Townsend deprivation index, alcohol status, and baseline CVD status. a: *P* <0.05, b: *P* <0.01, c: *P* <0.001.

| Table S8. Hazard ratios (95% confidence intervals) of LE8 metrics per 10 points increment associated with individual cancer risk in women | | | | | | | | |  |
| --- | --- | --- | --- | --- | --- | --- | --- | --- | --- |
| **Cancer Type** | **Blood pressure score** | **Sleep health score** | **Body mass index score** | **Tobacco/nicotine exposure score** | **Physical activity score** | **Blood lipid score** | **Blood glucose score** | **Diet score** |  |
| Overall | 1 (1-1) | 1 (1-1.01) | 0.99 (0.99-1) ^b^ | 0.98 (0.98-0.98) ^c^ | 0.99 (0.99-1) ^c^ | 1 (1-1.01) | 0.99 (0.98-1) ^b^ | 0.99 (0.99-1) ^c^ |  |
| Oral | 1.01 (0.97-1.06) | 1.06 (0.98-1.14) | 1.02 (0.97-1.07) | 0.89 (0.86-0.92) ^c^ | 0.96 (0.93-1) a | 0.97 (0.92-1.01) | 0.96 (0.89-1.02) | 0.98 (0.94-1.03) |  |
| Esophageal | 1.01 (0.96-1.06) | 1 (0.92-1.08) | 1 (0.95-1.06) | 0.93 (0.89-0.97) ^c^ | 0.97 (0.94-1.01) | 1.03 (0.98-1.09) | 0.97 (0.9-1.04) | 0.97 (0.92-1.02) |  |
| EAC | 0.94 (0.87-1.03) | 0.98 (0.86-1.11) | 0.9 (0.83-0.97) ^b^ | 0.92 (0.86-0.99) ^a^ | 0.96 (0.9-1.02) | 1.08 (1-1.17) | 0.91 (0.82-1.02) | 1 (0.92-1.09) |  |
| ESCC | 1.05 (0.98-1.12) | 1.01 (0.91-1.12) | 1.15 (1.06-1.25) ^c^ | 0.94 (0.89-1) ^a^ | 0.99 (0.94-1.05) | 1 (0.93-1.07) | 1.07 (0.96-1.21) | 0.98 (0.91-1.05) |  |
| Stomach | 1 (0.95-1.05) | 1 (0.92-1.08) | 0.96 (0.91-1.02) | 0.99 (0.94-1.03) | 0.99 (0.95-1.04) | 0.99 (0.94-1.05) | 0.99 (0.91-1.07) | 0.99 (0.94-1.05) |  |
| Small intestine | 1.02 (0.95-1.09) | 0.98 (0.88-1.09) | 0.96 (0.89-1.03) | 1.08 (1.01-1.16) ^a^ | 1 (0.94-1.06) | 1.01 (0.94-1.08) | 0.96 (0.86-1.07) | 0.98 (0.91-1.06) |  |
| Colorectal | 1 (0.98-1.01) | 1.01 (0.99-1.04) | 0.99 (0.97-1) | 0.97 (0.96-0.99) ^c^ | 1.01 (0.99-1.02) | 0.99 (0.97-1.01) | 0.97 (0.94-0.99) ^b^ | 0.98 (0.97-1) ^a^ |  |
| Anus | 1.08 (1-1.16) ^a^ | 0.99 (0.88-1.11) | 1.01 (0.94-1.1) | 0.93 (0.87-0.99) ^a^ | 0.99 (0.93-1.06) | 1.01 (0.93-1.09) | 0.94 (0.84-1.05) | 1.06 (0.98-1.14) |  |
| Liver | 1 (0.94-1.06) | 0.95 (0.87-1.03) | 0.95 (0.89-1) | 0.94 (0.89-0.98) ^b^ | 0.98 (0.94-1.03) | 1.01 (0.95-1.07) | 0.96 (0.88-1.04) | 0.96 (0.91-1.02) |  |
| HCC | 1.03 (0.91-1.16) | 0.86 (0.74-1.01) | 0.87 (0.77-0.98) ^a^ | 0.94 (0.85-1.05) | 0.95 (0.87-1.05) | 1.09 (0.96-1.23) | 0.92 (0.77-1.1) | 0.97 (0.86-1.1) |  |
| CAC | 0.98 (0.92-1.05) | 0.97 (0.88-1.07) | 0.98 (0.91-1.05) | 0.93 (0.88-0.99) ^a^ | 1 (0.95-1.06) | 0.99 (0.93-1.06) | 0.95 (0.86-1.05) | 0.96 (0.9-1.03) |  |
| Pancreatic | 0.98 (0.95-1.02) | 0.99 (0.94-1.04) | 0.93 (0.9-0.97) ^c^ | 0.95 (0.92-0.98) ^c^ | 0.99 (0.96-1.01) | 1.03 (0.99-1.06) | 0.89 (0.85-0.93) ^c^ | 1 (0.96-1.03) |  |
| Laryngeal | 0.99 (0.74-1.33) | 1.74 (0.52-5.85) | 0.93 (0.7-1.23) | 0.92 (0.72-1.18) | 1.41 (0.58-3.43) | 1.2 (0.9-1.6) | 0.99 (0.6-1.63) | 0.87 (0.65-1.16) |  |
| Lung | 1.02 (1-1.04) ^a^ | 0.95 (0.92-0.97) ^c^ | 1.03 (1.01-1.05) ^c^ | 0.75 (0.74-0.77) ^c^ | 0.97 (0.96-0.98) ^c^ | 1.02 (1-1.04) | 0.94 (0.92-0.97) ^c^ | 0.92 (0.9-0.94) ^c^ |  |
| SCLC | 1.02 (0.98-1.08) | 0.98 (0.91-1.05) | 0.98 (0.93-1.03) | 0.74 (0.7-0.78) ^c^ | 0.98 (0.94-1.01) | 0.97 (0.93-1.02) | 0.93 (0.87-0.99) ^a^ | 0.89 (0.85-0.94) ^c^ |  |
| NSCLC | 1.01 (0.99-1.04) | 0.94 (0.92-0.97) ^c^ | 1.05 (1.02-1.07) ^c^ | 0.76 (0.74-0.77) ^c^ | 0.98 (0.96-0.99) ^b^ | 1.02 (1-1.04) ^a^ | 0.95 (0.92-0.98) ^b^ | 0.93 (0.91-0.95) ^c^ |  |
| Melanoma skin | 1.01 (1-1.02) ^b^ | 1.02 (1-1.03) ^a^ | 1.04 (1.03-1.05) ^c^ | 1.01 (1-1.02) ^b^ | 1 (1-1.01) | 1.01 (1-1.01) | 1.04 (1.02-1.05) ^c^ | 1.01 (1-1.02) ^b^ |  |
| Mesothelioma | 1.04 (0.95-1.14) | 1.07 (0.91-1.25) | 1.04 (0.94-1.14) | 0.94 (0.87-1.02) | 0.97 (0.91-1.04) | 1 (0.91-1.09) | 0.99 (0.86-1.13) | 1.01 (0.92-1.11) |  |
| Soft tissue | 1 (0.95-1.05) | 0.96 (0.89-1.04) | 0.96 (0.91-1.01) | 1.01 (0.97-1.06) | 0.99 (0.95-1.03) | 1 (0.95-1.05) | 0.94 (0.87-1.02) | 1.01 (0.95-1.06) |  |
| Breast | 0.99 (0.98-1) ^b^ | 1.01 (1-1.03) ^b^ | 0.98 (0.97-0.99) ^c^ | 0.99 (0.98-1) ^a^ | 0.99 (0.98-0.99) ^c^ | 1 (0.99-1.01) | 1 (0.99-1.02) | 0.99 (0.98-0.99) ^c^ |  |
| Uterine | 0.96 (0.94-0.98) ^c^ | 1 (0.96-1.03) | 0.83 (0.82-0.85) ^c^ | 1.05 (1.03-1.07) ^c^ | 0.97 (0.96-0.99) ^b^ | 0.98 (0.96-1) | 0.89 (0.86-0.91) ^c^ | 1 (0.98-1.02) |  |
| Ovarian | 1.01 (0.98-1.03) | 1 (0.96-1.04) | 0.98 (0.95-1) | 0.99 (0.97-1.01) | 1 (0.98-1.03) | 1 (0.97-1.02) | 1.02 (0.98-1.07) | 1.01 (0.98-1.03) |  |
| Kidney | 0.94 (0.9-0.98) ^b^ | 1 (0.94-1.06) | 0.92 (0.89-0.95) ^c^ | 0.96 (0.93-0.99) ^b^ | 0.97 (0.94-1) ^a^ | 0.99 (0.95-1.03) | 0.92 (0.87-0.97) ^b^ | 0.98 (0.95-1.02) |  |
| RCC | 0.95 (0.91-0.99) ^a^ | 0.98 (0.92-1.04) | 0.92 (0.88-0.95) ^c^ | 0.97 (0.94-1) | 0.97 (0.94-1) ^a^ | 0.98 (0.94-1.02) | 0.93 (0.88-0.98) ^a^ | 0.98 (0.95-1.03) |  |
| TCC | 0.78 (0.65-0.94) ^b^ | 1.15 (0.86-1.55) | 0.96 (0.83-1.11) | 0.86 (0.76-0.98) ^a^ | 0.97 (0.86-1.08) | 1.21 (1.04-1.4) ^a^ | 0.88 (0.72-1.07) | 0.93 (0.8-1.08) |  |
| Bladder | 1.03 (0.98-1.09) | 1.04 (0.96-1.13) | 0.97 (0.93-1.02) | 0.95 (0.91-0.99) | 0.97 (0.94-1.01) | 0.98 (0.93-1.03) | 0.96 (0.89-1.03) | 1.01 (0.95-1.06) |  |
| Brain | 1 (0.96-1.05) | 1.01 (0.94-1.09) | 1.01 (0.96-1.06) | 1 (0.97-1.04) | 0.98 (0.95-1.02) | 1.02 (0.98-1.07) | 1.01 (0.94-1.09) | 1.04 (0.99-1.09) |  |
| Thyroid | 0.98 (0.93-1.02) | 1 (0.93-1.07) | 0.96 (0.92-1.01) | 1.03 (0.99-1.07) | 1.01 (0.97-1.05) | 1.02 (0.98-1.07) | 0.96 (0.89-1.03) | 0.98 (0.94-1.03) |  |
| Lymphoma | 1.03 (1-1.05) ^a^ | 1 (0.96-1.04) | 0.99 (0.97-1.02) | 1 (0.98-1.02) | 0.99 (0.97-1.01) | 1.02 (1-1.05) | 0.98 (0.94-1.02) | 1.02 (0.99-1.04) |  |
| HL | 1 (0.9-1.1) | 1.11 (0.92-1.34) | 0.97 (0.87-1.07) | 1.04 (0.95-1.14) | 0.97 (0.9-1.04) | 0.92 (0.83-1.02) | 0.98 (0.84-1.15) | 0.96 (0.87-1.06) |  |
| NHL | 1.03 (1-1.06) ^a^ | 0.99 (0.95-1.04) | 1 (0.97-1.02) | 1.01 (0.98-1.03) | 1 (0.98-1.02) | 1.02 (1-1.05) | 0.99 (0.95-1.03) | 1.04 (1.01-1.07) ^a^ |  |
| Multiple myeloma | 1 (0.96-1.04) | 0.95 (0.9-1.01) | 0.98 (0.94-1.02) | 0.97 (0.93-1) | 1.02 (0.98-1.05) | 1.05 (1-1.09) ^a^ | 0.96 (0.9-1.02) | 0.99 (0.94-1.03) |  |
| Leukemia | 1 (0.96-1.03) | 0.96 (0.91-1.02) | 0.98 (0.94-1.02) | 1 (0.97-1.03) | 1.02 (0.99-1.05) | 1.03 (0.99-1.06) | 1.02 (0.96-1.09) | 0.96 (0.93-1) ^a^ |  |

HRs are calculated per 10-point increment in the score. LE8, Life's Essential 8; EAC, esophageal adenocarcinoma; ESCC, esophageal squamous cell carcinoma; HCC, hepatocellular carcinoma; CAC, cholangiocarcinoma; SCLC, small cell carcinoma; NSCLC, non-small cell carcinoma; RCC, renal cell carcinoma; TCC, transitional cell carcinoma; HL, Hodgkin's lymphoma; NHL, non-Hodgkin's lymphoma.

Models were fully adjusted with age, ethnicity, education level, annual household income, Townsend deprivation index, alcohol status, and baseline CVD and menstrual status. a: *P* <0.05, b: *P* <0.01, c: *P* <0.001.

| Table S9. The association between per 10 points increment of CVH and the risk of cancer mortality in men and women | | | | | | | |
| --- | --- | --- | --- | --- | --- | --- | --- |
| **Mortality Type** | **Men** | | | **Women** | | | ***P* for interaction** |
|  | **No. of cases** | **$est 10 years** | **HR (95%CI)** | **No. of cases** | **$est 10 years** | **HR (95%CI)** |  |
| All | 16034 | 5.53E-02(5.42E-02-5.64E-02) | 0.78 (0.77-0.79) ^c^ | 9728 | 2.89E-02(2.81E-02-2.97E-02) | 0.8 (0.78-0.81) ^c^ | 0.017 |
| Non-cancer | 9164 | 2.94E-02(2.86-02-3.02E-02) | 0.77 (0.75-0.78) ^c^ | 4620 | 1.17E-02(1.12E-02-1.22E-02) | 0.75 (0.73-0.77) ^c^ | 0.039 |
| Cancer | 6734 | 2.59E-02(2.51E-02-2.67E-02) | 0.8 (0.78-0.82) ^c^ | 5050 | 1.72E-02(1.66E-02-1.78E-02) | 0.84 (0.82-0.86) ^c^ | <0.001 |
| Oral | 117 | 3.61E-04(2.67E-04-4.55E-04) | 0.77 (0.66-0.89) ^c^ | 36 | 1.32E-04(7.54E-05-1.89E-04) | 0.86 (0.66-1.12) | 0.227 |
| Esophageal | 455 | 1.76E-03(1.55E-03-1.97E-03) | 0.73 (0.67-0.78) ^c^ | 154 | 4.66E-04(3.60E-04-5.72E-04) | 0.88 (0.77-1) | 0.006 |
| Stomach | 220 | 8.61E-04(7.17E-04-1.01E-03) | 0.84 (0.75-0.94) ^b^ | 110 | 4.43E-04(3.39E-04-5.47E-04) | 0.86 (0.74-1) | 0.658 |
| Small intestine | 30 | 1.20E-04(6.60E-05-1.74E-04) | 0.77 (0.57-1.03) | 21 | 8.06E-05(3.64E-05-1.25E-04) | 1.33 (0.9-1.96) | 0.027 |
| Colorectal | 704 | 2.75E-03(2.49E-03-3.01E-03) | 0.86 (0.81-0.92) ^c^ | 509 | 1.69E-03(1.49E-03-1.89E-03) | 0.9 (0.83-0.96) ^b^ | 0.462 |
| Anus | 10 | 3.80E-05(7.64E-06-6.84E-05) | 0.59 (0.36-0.96) ^a^ | 14 | 2.88E-05(2.37E-06-5.52E-05) | 0.71 (0.47-1.07) | 0.267 |
| Liver | 305 | 1.05E-03(8.90E-04-1.21E-03) | 0.63 (0.58-0.69) ^c^ | 173 | 6.22E-04(4.99E-04-7.45E-04) | 0.75 (0.66-0.84) ^c^ | 0.008 |
| Pancreatic | 586 | 2.22E-03(1.99E-03-2.45E-03) | 0.86 (0.8-0.92) ^c^ | 449 | 1.50E-03(1.31E-03-1.69E-03) | 0.85 (0.79-0.92) ^c^ | 0.977 |
| Laryngeal | 24 | 7.60E-05(3.31E-05-1.19E-04) | 0.51 (0.37-0.7) ^c^ | 3 | 5.76E-06(0-1.76E-05) | 1.22 (0.43-3.43) | 0.091 |
| Lung | 1222 | 5.10E-03(4.75E-03-5.45E-03) | 0.67 (0.64-0.7) ^c^ | 947 | 3.35E-03(3.07E-03-3.63E-03) | 0.65 (0.61-0.68) ^c^ | 0.45 |
| Melanoma skin | 142 | 5.44E-04(4.29E-04-6.59E-04) | 0.96 (0.83-1.1) | 82 | 2.36E-04(1.60E-04-3.12E-04) | 0.93 (0.78-1.12) | 0.874 |
| Mesothelioma | 205 | 9.87E-04(8.32E-04-1.14E-03) | 1.04 (0.92-1.17) | 45 | 2.19E-04(1.46E-04-2.92E-04) | 0.96 (0.75-1.23) | 0.697 |
| Soft tissue | 55 | 2.41E-04(1.65E-04-3.17E-04) | 0.93 (0.75-1.17) | 104 | 4.20E-04(3.19E-04-5.21E-04) | 0.89 (0.76-1.04) | 0.976 |
| Breast | 6 | 2.53E-05(5.24E-07-5.01E-05) | 0.91 (0.47-1.76) | 500 | 1.73E-03(1.53E-03-1.93E-03) | 0.9 (0.84-0.97) ^b^ | 0.697 |
| Uterine | NA | NA | NA | 187 | 6.27E-04(5.04E-04-7.50E-04) | 0.92 (0.81-1.03) |  |
| Ovarian | NA | NA | NA | 390 | 1.33E-03(1.15E-03-1.51E-03) | 0.99 (0.91-1.07) |  |
| Prostate | 619 | 2.04E-03(1.82E-03-2.26E-03) | 0.89 (0.83-0.95) ^c^ | NA | NA | NA |  |
| Kidney | 229 | 9.37E-04(7.86E-04-1.09E-03) | 0.73 (0.66-0.82) ^c^ | 86 | 3.05E-04(2.19E-04-3.91E-04) | 0.77 (0.65-0.91) ^b^ | 0.544 |
| Bladder | 216 | 7.53E-04(6.18E-04-8.88E-04) | 0.74 (0.67-0.83) ^c^ | 67 | 2.13E-04(1.41E-04-2.85E-04) | 0.7 (0.58-0.84) ^c^ | 0.373 |
| Brain | 337 | 1.44E-03(1.25E-03-1.63E-03) | 0.98 (0.89-1.07) | 247 | 8.46E-04(7.03E-04-9.89E-04) | 1.04 (0.93-1.16) | 0.489 |
| Thyroid | 12 | 5.07E-05(1.56E-05-8.58E-05) | 0.76 (0.47-1.23) | 16 | 2.88E-05(2.37E-06-5.52E-05) | 0.82 (0.54-1.25) | 0.813 |
| Lymphoma | 286 | 1.03E-03(8.72E-04-1.19E-03) | 0.92 (0.84-1.02) | 175 | 5.35E-04(4.21E-04-6.49E-04) | 1.02 (0.9-1.16) | 0.251 |
| Multiple myeloma | 142 | 3.99E-04(3.01E-04-4.97E-04) | 0.87 (0.75-1) ^a^ | 90 | 2.76E-04(1.94E-04-3.58E-04) | 0.81 (0.68-0.96) ^a^ | 0.305 |
| Leukemia | 238 | 9.30E-04(7.80E-04-1.08E-03) | 0.92 (0.83-1.03) | 133 | 4.37E-04(3.34E-04-5.40E-04) | 0.96 (0.83-1.11) | 0.489 |

CVH, cardiovascular disease health; Models were fully adjusted with age, ethnicity, education level, annual household income, Townsend deprivation index, alcohol status, and baseline CVD status. Additionally, for women, the model was further adjusted menopausal status. $est: represents the estimated cumulative incidence rate, a: *P* <0.05, b: *P* <0.01, c: *P* <0.001.

| Table S10. Associations between CVH levels and risk of cancer mortality in men and women | | | | | | |  |
| --- | --- | --- | --- | --- | --- | --- | --- |
| **Cancer site** | **Men** | | | **Women** | | |  |
|  | **Ref** | **HR (95% CI)** | **HR (95% CI)** | **Ref** | **HR (95% CI)** | **HR (95% CI)** |  |
|  | **Low** | **Moderate** | **High** | **Low** | **Moderate** | **High** |  |
| All | 1 | 0.59 (0.57-0.62) ^c^ | 0.38 (0.35-0.41) ^c^ | 1 | 0.54 (0.51-0.57) ^c^ | 0.41 (0.37-0.44) ^c^ |  |
| Non-cancer | 1 | 0.56 (0.53-0.58) ^c^ | 35 (0.32-0.39) ^c^ | 1 | 0.49 (0.45-0.53) ^c^ | 0.33 (0.3-0.38) ^c^ |  |
| Cancer | 1 | 0.65 (0.61-0.69) ^c^ | 0.41 (0.36-0.46) ^c^ | 1 | 0.6 (0.56-0.66) ^c^ | 0.49 (0.43-0.54) |  |
| Oral | 1 | 0.72 (0.46-1.12) | 0.08 (0.01-0.59) ^a^ | 1 | 0.38 (0.16-0.91) ^a^ | 0.33 (0.1-1.09) |  |
| Esophageal | 1 | 0.67 (0.53-0.84) ^c^ | 0.14 (0.07-0.31) ^c^ | 1 | 0.51 (0.32-0.79) ^b^ | 0.42 (0.23-0.78) ^b^ |  |
| Stomach | 1 | 0.8 (0.57-1.13) | 0.46 (0.22-0.95) ^a^ | 1 | 0.6 (0.35-1.04) | 0.41 (0.19-0.88) ^a^ |  |
| Small intestine | 1 | 0.66 (0.27-1.64) | 0.01 (0-1328.03) | 1 | NA | NA |  |
| Colorectal | 1 | 0.72 (0.59-0.88) ^b^ | 0.54 (0.38-0.77) ^c^ | 1 | 0.65 (0.49-0.86) ^b^ | 0.61 (0.43-0.87) ^b^ |  |
| Anus | 1 | 0.34 (0.09-1.24) | NA | 1 | 0.52 (0.11-2.37) | 0.19 (0.02-2.23) |  |
| Liver | 1 | 0.39 (0.3-0.5) ^c^ | 0.2 (0.11-0.39) ^c^ |  | 0.69 (0.43-1.08) | 0.35 (0.18-0.68) ^b^ |  |
| Pancreatic | 1 | 0.74 (0.6-0.92) ^b^ | 0.55 (0.37-0.82) ^b^ | 1 | 0.54 (0.41-0.71) ^c^ | 0.45 (0.31-0.65) ^c^ |  |
| Laryngeal | 1 | 0.23 (0.1-0.54) ^c^ | NA | 1 | NA | NA |  |
| Lung | 1 | 0.5 (0.44-0.56) ^c^ | 0.16 (0.1-0.25) | 1 | 0.4 (0.34-0.47) ^c^ | 0.16 (0.12-0.22) ^c^ |  |
| Melanoma skin | 1 | 0.71 (0.45-1.11) | 0.97 (0.5-1.88) | 1 | 2.16 (0.68-6.92) | 1.94 (0.55-6.85) |  |
| Mesothelioma | 1 | 0.96 (0.65-1.41) | 0.93 (0.48-1.79) | 1 | 0.68 (0.28-1.64) | 0.43 (0.12-1.58) |  |
| Soft tissue | 1 | 0.88 (0.42-1.82) | 0.4 (0.09-1.89) | 1 | 0.77 (0.39-1.49) | 0.71 (0.32-1.58) |  |
| Breast | 1 | 0.32 (0.05-1.97) | 1.22 (0.1-14.42) | 1 | 0.83 (0.6-1.14) | 0.66 (0.45-0.97) |  |
| Uterine | 1 | NA | NA |  | 0.51 (0.33-0.78) ^b^ | 0.46 (0.27-0.81) ^b^ |  |
| Ovarian | 1 | NA | NA | 1 | 0.96 (0.66-1.38) | 0.98 (0.64-1.51) |  |
| Prostate | 1 | 0.76 (0.62-0.94) ^a^ | 0.61 (0.42-0.9) ^a^ | 1 | NA | NA |  |
| Kidney | 1 | 0.57 (0.42-0.78) ^c^ | 0.21 (0.09-0.49) ^c^ | 1 | 0.49 (0.27-0.89) ^a^ | 0.21 (0.08-0.57) ^b^ |  |
| Bladder | 1 | 0.64 (0.46-0.9) ^b^ | 0.34 (0.16-0.73) ^b^ | 1 | 0.51 (0.27-0.98) ^a^ | 0.27 (0.09-0.79) ^a^ |  |
| Brain | 1 | 0.9 (0.66-1.22) | 0.85 (0.52-1.37) | 1 | 1.23 (0.74-2.07) | 1.34 (0.75-2.39) |  |
| Thyroid | 1 | 0.44 (0.12-1.68) | NA (-) | 1 | 0.48 (0.1-2.21) | 0.63 (0.1-3.93) |  |
| Lymphoma | 1 | 0.94 (0.68-1.31) | 0.69 (0.38-1.23) | 1 | 0.81 (0.49-1.36) | 1.08 (0.59-1.97) |  |
| Multiple myeloma | 1 | 0.78 (0.49-1.24) | 0.77 (0.37-1.6) | 1 | 0.49 (0.27-0.88) ^a^ | 0.44 (0.2-0.98) ^a^ |  |
| Leukemia | 1 | 0.71 (0.51-1) ^a^ | 0.65 (0.37-1.17) | 1 | 1.62 (0.75-3.49) | 1.04 (0.42-2.59) |  |

HRs are calculated per 10-point increment in the CVH score. CVH, cardiovascular disease health; Models were fully adjusted with age, ethnicity, education level, annual household income, Townsend deprivation index, alcohol status, and baseline CVD status. Additionally, for women, the model was further adjusted menopausal status. a: *P* <0.05, b: *P* <0.01, c: *P* <0.001.

| Table S11. Subgroup analysis of the correlation between per 10-point increment in CVH score in clinical variables and cancer risk | | | | | | |
| --- | --- | --- | --- | --- | --- | --- |
| **Subgroups** | **Total** | ***P* for interaction** | **Men** | ***P* for interaction** | **Women** | ***P* for interaction** |
| **Age** |  | 0.251 |  | 0.269 |  | 0.746 |
| <60 | 0.97 (0.96-0.98) ^c^ |  | 0.97 (0.96-0.99) ^b^ |  | 0.97 (0.96-0.99) ^c^ |  |
| ≥60 | 0.97 (0.96-0.98) ^c^ |  | 0.98 (0.97-0.99) ^c^ |  | 0.95 (0.94-0.97) ^c^ |  |
| **Ethnicity** |  | 0.135 |  | 0.127 |  | 0.651 |
| white | 0.97 (0.96-0.98) ^c^ |  | 0.97 (0.96-0.98) ^c^ |  | 0.96 (0.95-0.97) ^c^ |  |
| others | 0.94 (0.9-0.99) ^a^ |  | 0.95 (0.89-1.01) |  | 0.94 (0.88-1) |  |
| **CVD** |  | 0.149 |  | 0.042 |  | 0.013 |
| No | 0.97 (0.97-0.98) ^c^ |  | 0.98 (0.97-0.99) ^c^ |  | 0.97 (0.95-0.98) ^c^ |  |
| Yes | 0.95 (0.93-0.97) ^c^ |  | 0.95 (0.93-0.98) ^c^ |  | 0.92 (0.89-0.96) ^c^ |  |
| **Townsend deprivation index** |  | 0.023 |  | 0.068 |  | 0.122 |
| Low | 0.98 (0.97-0.99) ^c^ |  | 0.98 (0.97-0.99) ^b^ |  | 0.98 (0.96-0.99) ^c^ |  |
| High | 0.96 (0.94-0.97) ^c^ |  | 0.97 (0.95-0.98) ^c^ |  | 0.94 (0.93-0.96) ^c^ |  |
| **Education** |  | 0.121 |  | 0.137 |  | 0.084 |
| College/university degree | 0.97 (0.96-0.98) ^c^ |  | 0.98 (0.97-1) ^a^ |  | 0.95 (0.94-0.97) ^c^ |  |
| Under college/university degree | 0.97 (0.96-0.98) ^c^ |  | 0.97 (0.96-0.98) ^c^ |  | 0.97 (0.96-0.98) ^c^ |  |
| Unknown | 0.91 (0.84-0.98) ^a^ |  | 0.95 (0.86-1.05) |  | 0.86 (0.76-0.96) ^b^ |  |
| **Annual household income** |  | 0.067 |  | <0.001 |  | 0.426 |
| low | 0.96 (0.95-0.97) ^c^ |  | 0.96 (0.95-0.98) ^c^ |  | 0.95 (0.94-0.97) ^c^ |  |
| high | 0.98 (0.97-0.99) ^b^ |  | 0.99 (0.97-1) |  | 0.97 (0.95-0.99) ^b^ |  |
| Unknown | 0.97 (0.95-0.99) ^b^ |  | 0.97 (0.94-1) |  | 0.96 (0.94-0.99) ^b^ |  |
| **Menopausal status** |  | 0.281 |  |  |  |  |
| No | 0.98 (0.96-1.01) |  | NA |  | 0.98 (0.96-1.01) | 0.281 |
| Yes | 0.96 (0.94-0.97) ^c^ |  | NA |  | 0.96 (0.94-0.97) ^c^ |  |
| Unknown | 0.96 (0.94-0.99) ^b^ |  | NA |  | 0.96 (0.94-0.99) ^b^ |  |
| **Alcohol status** |  | <0.001 |  | <0.001 |  | 0.009 |
| No | 0.96 (0.92-0.99) ^a^ |  | 0.99 (0.93-1.06) |  | 0.94 (0.9-0.98) ^b^ |  |
| Yes | 0.97 (0.96-0.98) ^c^ |  | 0.97 (0.96-0.98) ^c^ |  | 0.96 (0.95-0.97) ^c^ |  |
| Unknown | 1.15 (0.83-1.6) |  | 0.81 (0.49-1.33) |  | 1.52 (0.94-2.46) |  |

CVH, cardiovascular disease health; Townsend deprivation index: low and high was defined as less or high than mean value, respectively; Education: "college/university degree" refers to those with an ISCED level of 5, and “under college/university degree" refers to those with an ISCED level of 1-4. Annual household income: "low" indicates participants with an income of <£18,000 and £18,000-29,999, and "high" indicates participants with an income of £30,000-51,999, £52,000-100,000, and >£100,000; Alcohol status: "yes" refers to participants with an alcohol status of "previous" and "current," while "no" refers to participants with an alcohol status of "never."

Models were fully adjusted with age, ethnicity, education level, annual household income, Townsend deprivation index, alcohol status, and baseline CVD status. Additionally, for women, the model was further adjusted menopausal status. a: *P* <0.05, b: *P* <0.01, c: *P* <0.001.

| Table S12. The association between per 10 points increment of CVH and cancer risk with multiple imputations in men and women | | | | | | | | | | |
| --- | --- | --- | --- | --- | --- | --- | --- | --- | --- | --- |
| **Cancer Type** | Imputation 1 | | Imputation 2 | | Imputation 3 | | Imputation 4 | | Imputation 5 | |
|  | Men | Women | Men | Women | Men | Women | Men | Women | Men | Women |
|  | HR (95%CI) | HR (95%CI) | HR (95%CI) | HR (95%CI) | HR (95%CI) | HR (95%CI) | HR (95%CI) | HR (95%CI) | HR (95%CI) | HR (95%CI) |
| Overall | 0.97 (0.96,0.98) ^c^ | 0.96 (0.95,0.97) ^c^ | 0.97 (0.96,0.98) ^c^ | 0.96 (0.95,0.97) ^c^ | 0.97 (0.96,0.98) ^c^ | 0.96 (0.95,0.97) ^c^ | 0.97 (0.96,0.98) ^c^ | 0.96 (0.95,0.97) ^c^ | 0.97 (0.96,0.98) ^c^ | 0.96 (0.95,0.97) ^c^ |
| Oral | 0.82 (0.76,0.88) ^c^ | 0.83 (0.75,0.92) ^c^ | 0.82 (0.76,0.88) ^c^ | 0.83 (0.75,0.92) ^c^ | 0.82 (0.76,0.88) ^c^ | 0.84 (0.75,0.93) ^c^ | 0.82 (0.76,0.88) ^c^ | 0.83 (0.75,0.93) ^c^ | 0.82 (0.76,0.88) ^c^ | 0.83 (0.75,0.93) ^c^ |
| Esophageal | 0.71 (0.66,0.77) ^c^ | 0.89 (0.78,1) | 0.71 (0.66,0.77) ^c^ | 0.89 (0.78,1) | 0.71 (0.66,0.77) ^c^ | 0.88 (0.78,1) ^a^ | 0.71 (0.66,0.77) ^c^ | 0.89 (0.78,1) | 0.71 (0.66,0.77) ^c^ | 0.88 (0.78,1) ^a^ |
| EAC | 0.69 (0.64,0.76) ^c^ | 0.79 (0.65,0.96) ^a^ | 0.69 (0.64,0.76) ^c^ | 0.79 (0.65,0.96) ^a^ | 0.69 (0.64,0.76) ^c^ | 0.78 (0.64,0.95) ^a^ | 0.69 (0.64,0.76) ^c^ | 0.78 (0.64,0.95) ^a^ | 0.69 (0.64,0.76) ^c^ | 0.79 (0.65,0.96) ^a^ |
| ESCC | 0.78 (0.65,0.94) ^b^ | 1.05 (0.88,1.24) | 0.78 (0.65,0.94) ^b^ | 1.05 (0.88,1.24) | 0.79 (0.65,0.95) ^a^ | 1.05 (0.88,1.24) | 0.78 (0.65,0.95) ^b^ | 1.05 (0.88,1.25) | 0.78 (0.65,0.94) ^b^ | 1.04 (0.87,1.23) |
| Stomach | 0.79 (0.72,0.86) ^c^ | 0.93 (0.82,1.07) | 0.79 (0.72,0.86) ^c^ | 0.93 (0.82,1.07) | 0.79 (0.72,0.86) ^c^ | 0.93 (0.81,1.07) | 0.79 (0.72,0.86) ^c^ | 0.93 (0.81,1.07) | 0.79 (0.72,0.86) ^c^ | 0.93 (0.81,1.07) |
| Small intestine | 0.86 (0.73,1.03) | 1.02 (0.85,1.23) | 0.86 (0.72,1.03) | 1.01 (0.84,1.22) | 0.86 (0.73,1.03) | 1.02 (0.85,1.23) | 0.86 (0.72,1.03) | 1.01 (0.84,1.22) | 0.86 (0.73,1.03) | 1.02 (0.84,1.23) |
| Colorectal | 0.86 (0.83,0.89) ^c^ | 0.93 (0.9,0.97) ^b^ | 0.86 (0.83,0.89) ^c^ | 0.94 (0.9,0.97) ^b^ | 0.86 (0.83,0.89) ^c^ | 0.94 (0.9,0.97) ^b^ | 0.86 (0.83,0.89) ^c^ | 0.94 (0.9,0.97) ^b^ | 0.86 (0.83,0.89) ^c^ | 0.94 (0.9,0.97) ^b^ |
| Anus | 0.95 (0.75,1.2) | 1 (0.83,1.2) | 0.95 (0.75,1.2) | 1 (0.83,1.21) | 0.95 (0.75,1.2) | 1 (0.83,1.21) | 0.94 (0.75,1.19) | 1 (0.83,1.21) | 0.95 (0.75,1.2) | 1.01 (0.83,1.21) |
| Liver | 0.64 (0.58,0.7) ^c^ | 0.82 (0.71,0.95) ^b^ | 0.64 (0.58,0.7) ^c^ | 0.82 (0.71,0.94) ^b^ | 0.64 (0.58,0.7) ^c^ | 0.82 (0.72,0.95) ^b^ | 0.64 (0.58,0.7) ^c^ | 0.82 (0.71,0.94) ^b^ | 0.64 (0.58,0.7) ^c^ | 0.85 (0.71,0.95) ^b^ |
| HCC | 0.57 (0.51,0.64) ^c^ | 0.77 (0.57,1.05) | 0.57 (0.5,0.64) ^c^ | 0.77 (0.57,1.05) | 0.57 (0.51,0.64) ^c^ | 0.78 (0.57,1.05) | 0.57 (0.51,0.64) ^c^ | 0.77 (0.57,1.04) | 0.57 (0.51,0.64) ^c^ | 0.78 (0.57,1.05) |
| CAC | 0.81 (0.68,0.95) ^a^ | 0.84 (0.71,0.99) ^a^ | 0.81 (0.68,0.95) ^a^ | 0.84 (0.71,0.99) ^a^ | 0.81 (0.68,0.95) ^a^ | 0.84 (0.71,0.99) ^a^ | 0.81 (0.68,0.95) ^a^ | 0.83 (0.71,0.98) ^a^ | 0.81 (0.68,0.95) ^a^ | 0.84 (0.71,0.99) ^a^ |
| Pancreatic | 0.84 (0.78,0.91) ^c^ | 0.83 (0.77,0.9) c | 0.84 (0.78,0.91) ^c^ | 0.83 (0.76,0.9) ^c^ | 0.84 (0.78,0.91) ^c^ | 0.83 (0.77,0.9) ^c^ | 0.84 (0.78,0.91) ^c^ | 0.83 (0.77,0.9) ^c^ | 0.84 (0.78,0.91) ^c^ | 0.83 (0.77,0.9) ^c^ |
| Laryngeal | 0.65 (0.56,0.75) ^c^ | 1.1 (0.52,2.33) | 0.65 (0.56,0.75) ^c^ | 1.08 (0.51,2.27) | 0.65 (0.56,0.75) ^c^ | 1.08 (0.51,2.29) | 0.65 (0.56,0.75) ^c^ | 1.08 (0.51,2.29) | 0.65 (0.56,0.75) ^c^ | 1.07 (0.51,2.26) |
| Lung | 0.67 (0.64,0.7) ^c^ | 0.7 (0.67,0.73) ^c^ | 0.67 (0.64,0.7) ^c^ | 0.7 (0.67,0.73) ^c^ | 0.67 (0.64,0.7) ^c^ | 0.7 (0.67,0.73) ^c^ | 0.67 (0.64,0.7) ^c^ | 0.7 (0.67,0.73) ^c^ | 0.67 (0.64,0.7) ^c^ | 0.7 (0.67,0.73) ^c^ |
| SCLC | 0.64 (0.57,0.73) ^c^ | 0.64 (0.57,0.72) ^c^ | 0.65 (0.57,0.73) ^c^ | 0.64 (0.57,0.72) ^c^ | 0.64 (0.57,0.73) ^c^ | 0.64 (0.57,0.72) ^c^ | 0.64 (0.57,0.73) | 0.64 (0.57,0.72) ^c^ | 0.64 (0.57,0.73) ^c^ | 0.64 (0.57,0.72) ^c^ |
| NSCLC | 0.68 (0.65,0.71) ^c^ | 0.72 (0.68,0.76) ^c^ | 0.68 (0.65,0.72) ^c^ | 0.72 (0.68,0.76) ^c^ | 0.68 (0.65,0.71) ^c^ | 0.72 (0.68,0.76) ^c^ | 0.68 (0.65,0.72) ^c^ | 0.72 (0.68,0.76) ^c^ | 0.68 (0.65,0.71) ^c^ | 0.72 (0.68,0.76) ^c^ |
| Melanoma skin | 1.08 (1.06,1.1) ^c^ | 1.08 (1.06,1.11) ^c^ | 1.08 (1.06,1.1) ^c^ | 1.08 (1.06,1.11) ^c^ | 1.08 (1.06,1.1) ^c^ | 1.08 (1.06,1.11) ^c^ | 1.08 (1.06,1.1) ^c^ | 1.08 (1.06,1.1) ^c^ | 1.08 (1.06,1.1) ^c^ | 1.08 (1.06,1.1) ^c^ |
| Mesothelioma | 1.02 (0.91,1.15) | 0.97 (0.77,1.23) | 1.03 (0.91,1.15) | 0.97 (0.77,1.23) | 1.02 (0.91,1.15) | 0.97 (0.77,1.22) | 1.02 (0.91,1.15) | 0.97 (0.77,1.22) | 1.02 (0.91,1.15) | 0.97 (0.77,1.23) |
| Soft tissue | 0.99 (0.85,1.16) | 0.94 (0.83,1.07) | 0.99 (0.85,1.16) | 0.94 (0.83,1.07) | 0.99 (0.85,1.16) | 0.94 (0.83,1.07) | 0.99 (0.85,1.16) | 0.94 (0.83,1.07) | 0.99 (0.85,1.16) | 0.94 (0.83,1.07) |
| Breast | 0.92 (0.71,1.18) | 0.94 (0.92,0.96) ^c^ | 0.92 (0.71,1.17) | 0.94 (0.92,0.96) ^c^ | 0.91 (0.71,1.17) | 0.94 (0.92,0.96) ^c^ | 0.92 (0.72,1.18) | 0.94 (0.92,0.96) ^c^ | 0.91 (0.71,1.17) | 0.94 (0.92,0.96) ^c^ |
| Uterine | NA | 0.81 (0.77,0.85) ^c^ | NA | 0.81 (0.77,0.85) ^c^ | NA | 0.81 (0.77,0.85) ^c^ | NA | 0.81 (0.77,0.85) ^c^ | NA | 0.81 (0.77,0.85) ^c^ |
| Ovarian | NA | 0.99 (0.93,1.06) | NA | 0.99 (0.93,1.06) | NA | 0.99 (0.93,1.06) | NA | 0.99 (0.93,1.06) | NA | 0.99 (0.93,1.06) |
| Prostate | 1.05 (1.03,1.07) ^c^ | NA | 1.05 (1.03,1.07) ^c^ | NA | 1.05 (1.03,1.07) ^c^ | NA | 1.05 (1.03,1.07) ^c^ | NA | 1.05 (1.03,1.07) ^c^ | NA |
| Kidney | 0.82 (0.77,0.87) ^c^ | 0.78 (0.72,0.86) ^c^ | 0.82 (0.77,0.87) ^c^ | 0.79 (0.72,0.86) ^c^ | 0.82 (0.77,0.87) ^c^ | 0.78 (0.72,0.86) ^c^ | 0.82 (0.77,0.87) ^c^ | 0.79 (0.72,0.86) ^c^ | 0.82 (0.77,0.87) ^c^ | 0.78 (0.72,0.86) ^c^ |
| RCC | 0.84 (0.78,0.9) ^c^ | 0.79 (0.72,0.87) ^c^ | 0.84 (0.78,0.9) ^c^ | 0.79 (0.72,0.87) ^c^ | 0.84 (0.78,0.9) ^c^ | 0.79 (0.72,0.87) ^c^ | 0.84 (0.78,0.9) ^c^ | 0.79 (0.72,0.87) ^c^ | 0.84 (0.78,0.9) ^c^ | 0.79 (0.72,0.87) ^c^ |
| TCC | 0.77 (0.6,1) | 0.73 (0.51,1.05) | 0.77 (0.59,0.99) ^a^ | 0.72 (0.5,1.04) | 0.77 (0.6,1) ^a^ | 0.72 (0.51,1.03) | 0.77 (0.59,0.99) ^a^ | 0.73 (0.51,1.04) | 0.77 (0.59,0.99) ^a^ | 0.73 (0.51,1.05) |
| Bladder | 0.79 (0.74,0.85) ^c^ | 0.9 (0.8,1.02) | 0.79 (0.73,0.85) ^c^ | 0.91 (0.8,1.03) | 0.79 (0.73,0.85) ^c^ | 0.91 (0.8,1.03) | 0.79 (0.73,0.85) ^c^ | 0.91 (0.8,1.03) | 0.79 (0.73,0.85) ^c^ | 0.91 (0.8,1.03) |
| Brain | 0.95 (0.87,1.05) | 1.04 (0.93,1.16) | 0.95 (0.87,1.05) | 1.03 (0.92,1.16) | 0.96 (0.87,1.05) | 1.03 (0.92,1.16) | 0.95 (0.87,1.05) | 1.04 (0.93,1.16) | 0.96 (0.87,1.05) | 1.04 (0.92,1.16) |
| Thyroid | 0.94 (0.78,1.12) | 0.97 (0.87,1.09) | 0.93 (0.78,1.12) | 0.97 (0.87,1.09) | 0.93 (0.78,1.12) | 0.98 (0.87,1.09) | 0.94 (0.78,1.12) | 0.97 (0.87,1.09) | 0.93 (0.78,1.12) | 0.98 (0.87,1.09) |
| Lymphoma | 1.01 (0.95,1.07) | 1.03 (0.96,1.09) | 1.01 (0.95,1.07) | 1.03 (0.97,1.1) | 1.01 (0.95,1.07) | 1.03 (0.96,1.1) | 1.01 (0.95,1.07) | 1.03 (0.96,1.1) | 1.01 (0.95,1.07) | 1.03 (0.96,1.09) |
| HL | 0.89 (0.71,1.12) | 0.9 (0.7,1.15) | 0.89 (0.71,1.12) | 0.9 (0.7,1.15) | 0.89 (0.71,1.12) | 0.89 (0.69,1.14) | 0.89 (0.71,1.12) | 0.9 (0.7,1.15) | 0.89 (0.71,1.13) | 0.91 (0.71,1.17) |
| NHL | 1.03 (0.96,1.09) | 1.07 (1,1.15) | 1.03 (0.96,1.09) | 1.07 (1,1.15) ^a^ | 1.03 (0.96,1.09) | 1.07 (1,1.15) | 1.03 (0.96,1.09) | 1.07 (1,1.15) ^a^ | 1.03 (0.96,1.09) | 1.07 (1,1.15) |
| Multiple myeloma | 1 (0.91,1.09) | 0.96 (0.86,1.06) | 1 (0.91,1.09) | 0.96 (0.86,1.06) | 0.99 (0.91,1.09) | 0.96 (0.86,1.06) | 0.99 (0.91,1.09) | 0.96 (0.86,1.06) | 0.99 (0.91,1.09) | 0.96 (0.86,1.07) |
| Leukemia | 0.95 (0.89,1.03) | 0.98 (0.89,1.08) | 0.96 (0.89,1.03) | 0.98 (0.9,1.08) | 0.96 (0.89,1.03) | 0.98 (0.89,1.08) | 0.96 (0.89,1.03) | 0.98 (0.89,1.08) | 0.96 (0.89,1.03) | 0.98 (0.9,1.08) |

HRs are calculated per 10-point increment in the score. CVH, cardiovascular disease health; EAC, esophageal adenocarcinoma; ESCC, esophageal squamous cell carcinoma; HCC, hepatocellular carcinoma; CAC, cholangiocarcinoma; SCLC, small cell carcinoma; NSCLC, non-small cell carcinoma; RCC, renal cell carcinoma; TCC, transitional cell carcinoma; HL, Hodgkin's lymphoma; NHL, non-Hodgkin's lymphoma.

Models were fully adjusted with age, ethnicity, education level, annual household income, Townsend deprivation index, alcohol status, and baseline CVD status. Additionally, for women, the model was further adjusted menopausal status. a: *P* <0.05, b: *P* <0.01, c: *P* <0.001.


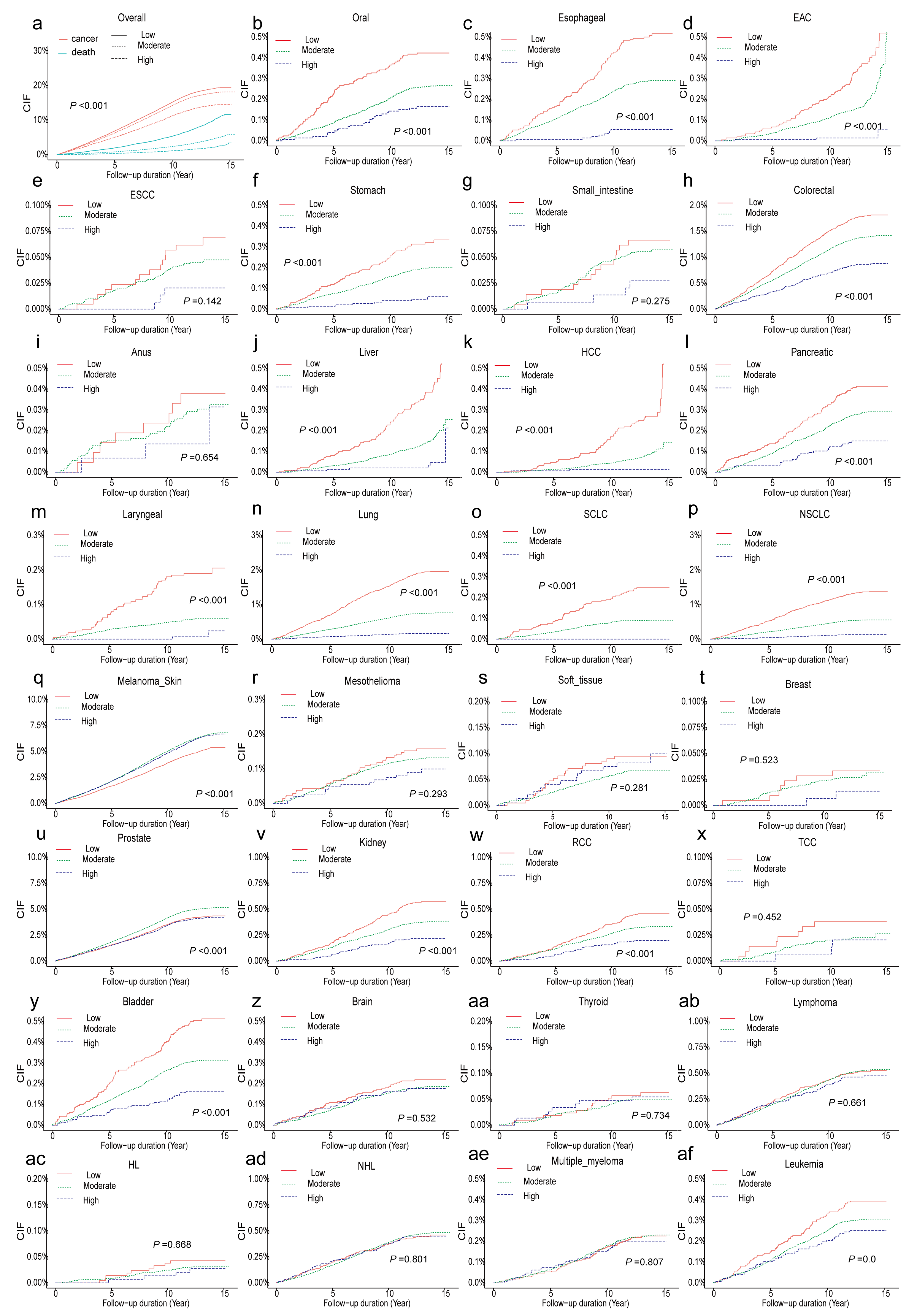


Fig. S1. Cumulative incidence of cancers according to CVH categories in men. (A) Overall cancer. (B) Oral cancer. (C) Esophageal cancer. (D) EAC. (E) ESCC. (F) Stomach cancer. (G) Small intestine cancer. (H) Colorectal cancer. (I) Anus cancer. (J) Liver cancer. (K) HCC. (L) Pancreatic cancer. (M) Laryngeal cancer. (N) Lung cancer. (O) SCLC. (P) NSCLC. (Q) Melanoma skin cancer. (R) Mesothelioma. (S) Soft tissue cancer. (T) Breast cancer. (U) Prostate cancer. (V) Kidney cancer. (W) RCC. (X) TCC. (Y) Bladder cancer. (Z) Brain cancer. (AA) Thyroid cancer. (AB) Lymphoma. (AC) HL. (AD) NHL. (AE) Multiple myeloma. (AF) Leukemia. CVH, cardiovascular health; EAC, esophageal adenocarcinoma; ESCC, esophageal squamous cell carcinoma; HCC, hepatocellular carcinoma; SCLC, small cell lung cancer; NSCLC, non-small cell lung cancer; RCC, renal cell carcinoma; TCC, transitional cell carcinoma; HL, Hodgkin's lymphoma; NHL, non-Hodgkin's lymphoma.


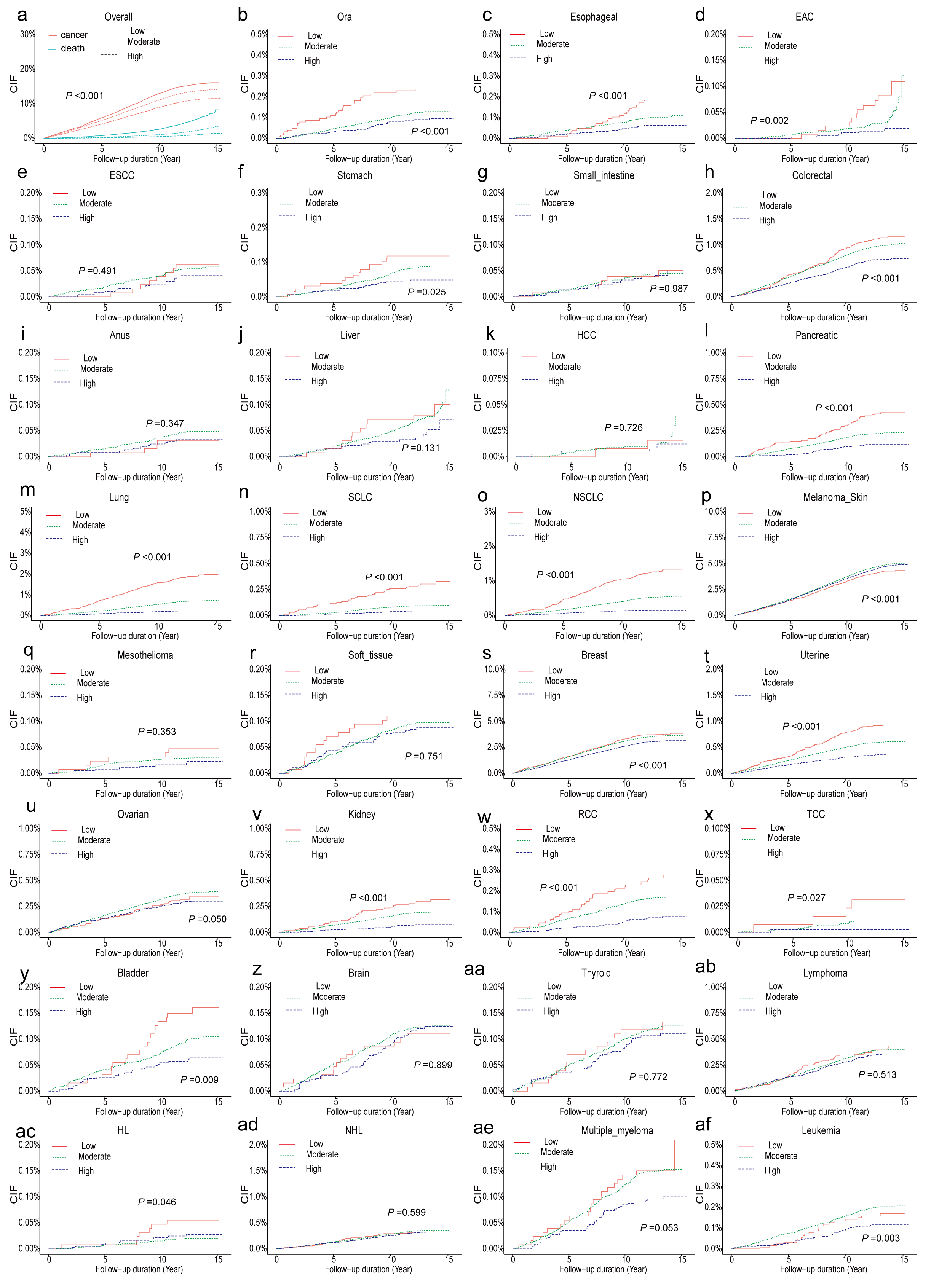


Fig. S2. Cumulative incidence of cancers according to CVH categories in women. (A) Overall cancer. (B) Oral cancer. (C) Esophageal cancer. (D) EAC. (E) ESCC. (F) Stomach cancer. (G) Small intestine cancer. (H) Colorectal cancer. (I) Anus cancer. (J) Liver cancer. (K) HCC. (L) Pancreatic cancer. (M) Lung cancer. (N) SCLC. (O) NSCLC. (P) Melanoma skin cancer. (Q) Mesothelioma. (R) Soft tissue cancer. (S) Breast cancer. (T) Uterine cancer. (U) Ovarian cancer. (V) Kidney cancer. (W) RCC. (X) TCC. (Y) Bladder cancer. (Z) Brain cancer. (AA) Thyroid cancer. (AB) Lymphoma. (AC) HL. (AD) NHL. (AE) Multiple myeloma. (AF) Leukemia. CVH, cardiovascular health; EAC, esophageal adenocarcinoma; ESCC, esophageal squamous cell carcinoma; HCC, hepatocellular carcinoma; SCLC, small cell lung cancer; NSCLC, non-small cell lung cancer; RCC, renal cell carcinoma; TCC, transitional cell carcinoma; HL, Hodgkin's lymphoma; NHL, non-Hodgkin's lymphoma.


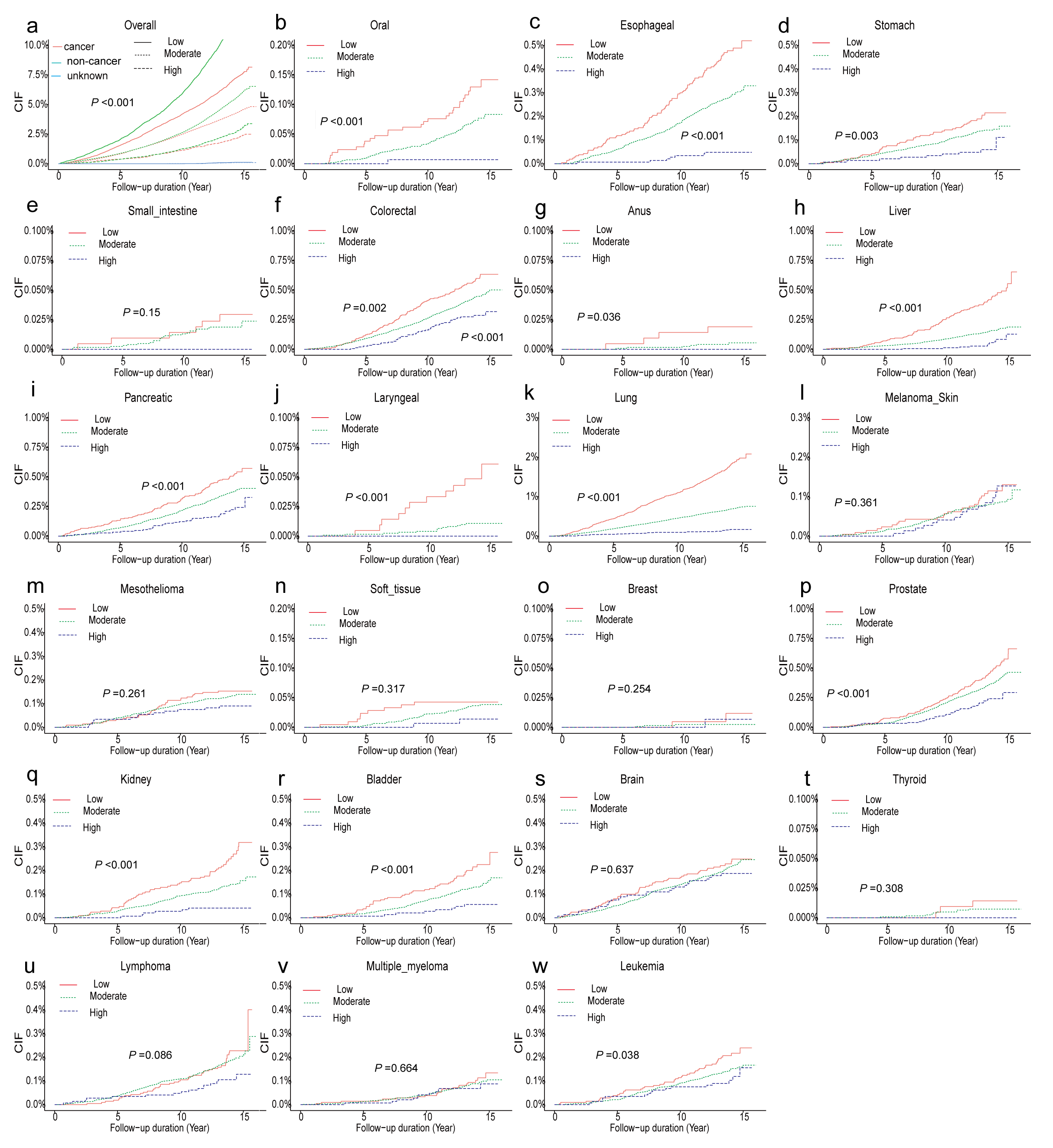


Fig. S3. Cumulative incidence curve of cancer mortality according to CVH categories in men. (A) Overall cancer. (B) Oral cancer. (C) Esophageal cancer. (D) Stomach cancer. (E) Small intestine cancer. (F) Colorectal cancer. (G) Anus cancer. (H) Liver cancer. (I) Pancreatic cancer. (J) Laryngeal cancer. (K) Lung cancer. (L) Melanoma skin cancer. (M) Mesothelioma. (N) Soft tissue cancer. (O) Breast cancer. (P) Prostate. (Q) Kidney cancer. (R) Bladder cancer. (S) Brain cancer. (T) Thyroid cancer. (U) Lymphoma. (V) Multiple myeloma. (W) Leukemia. CVH, cardiovascular health.


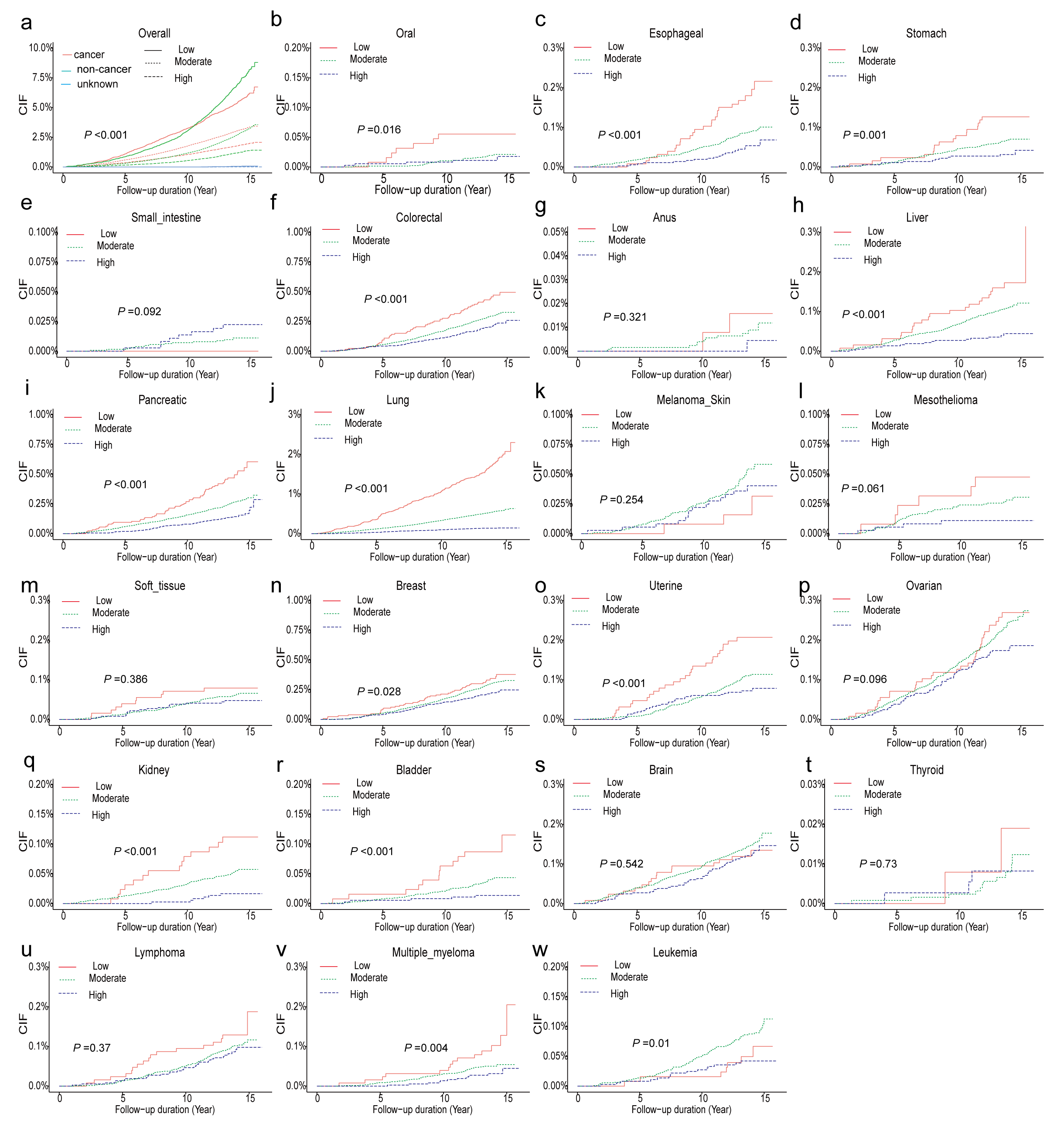


Fig. S4. Cumulative incidence curve of cancer mortality according to CVH categories in women. (A) Overall cancer. (B) Oral cancer. (C) Esophageal cancer. (D) Stomach cancer. (E) Small intestine cancer. (F) Colorectal cancer. (G) Anus cancer. (H) Liver cancer. (I) Pancreatic cancer. (J) Lung cancer. (K) Melanoma skin cancer. (L) Mesothelioma. (M) Soft tissue cancer. (N) Breast cancer. (O) Uterine cancer. (P) Ovarian cancer. (Q) Kidney cancer. (R) Bladder cancer. (S) Brain cancer. (T) Thyroid cancer. (U) Lymphoma. (V) Multiple myeloma. (W) Leukemia. CVH, cardiovascular health.


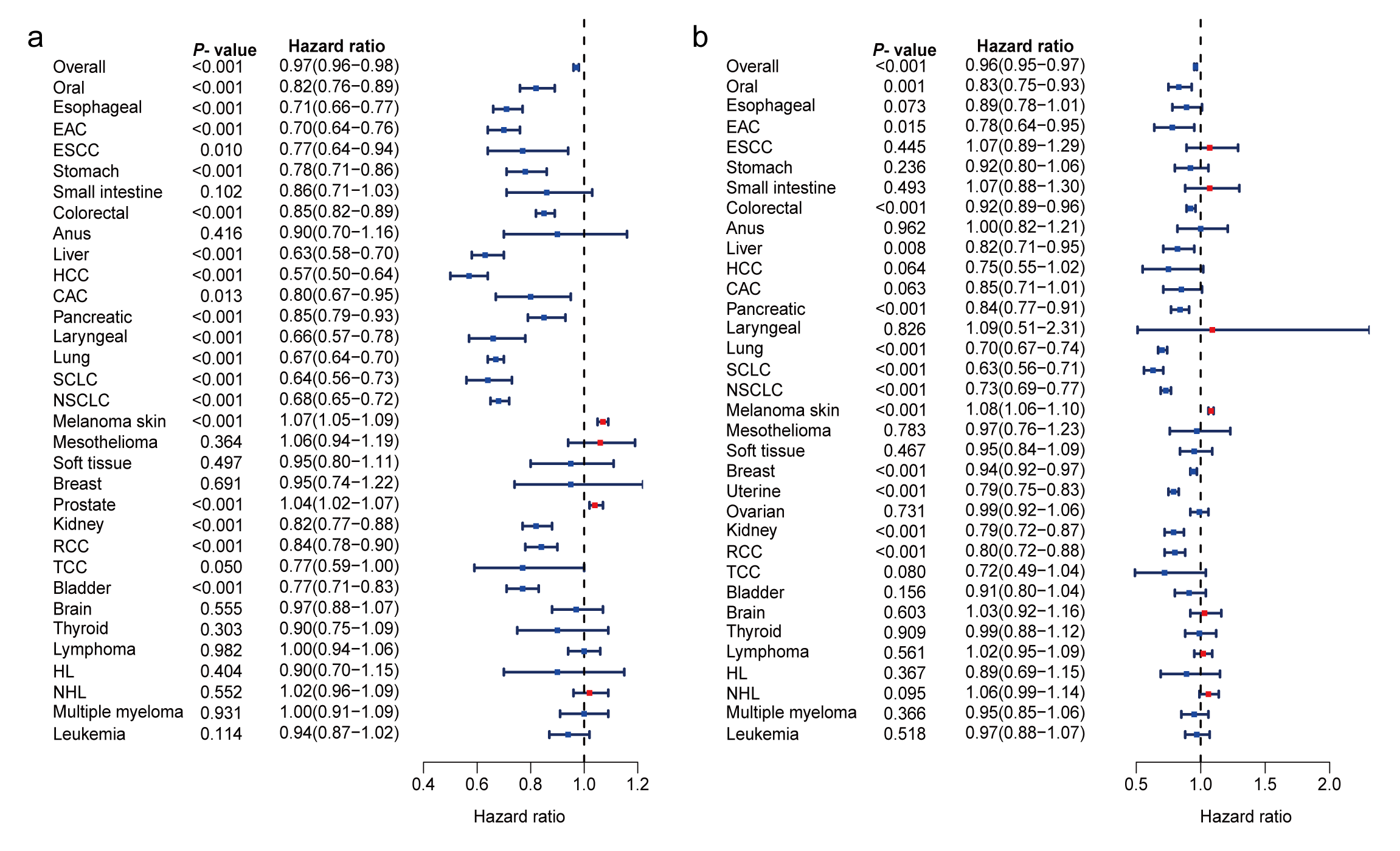


Fig. S5. Sensitivity analysis of the association between per 10-point increment in CVH score and the risk of cancer after excluding cancer events occurring within two years from the baseline in men (A) and women (B). Models were fully adjusted for age, ethnicity, education level, annual household income, Townsend deprivation index, alcohol status, and baseline CVD status. Additionally, for women, the model was adjusted for menopausal status. Participants with cancer that occurred during the initial two years of follow-up were excluded. CVH, cardiovascular health; CVD, cardiovascular disease. a: *P* <0.05, b: *P* <0.01, c: *P* <0


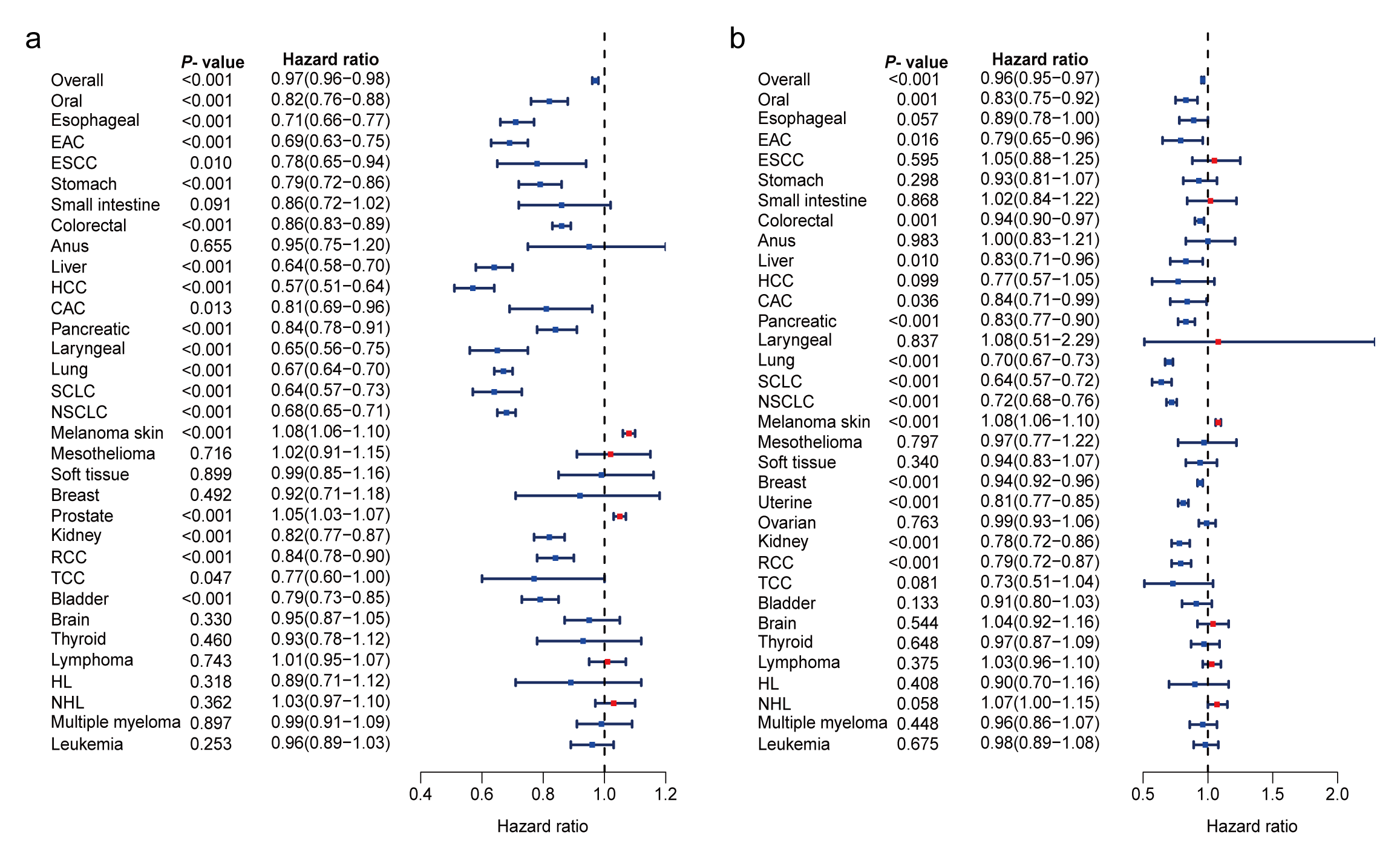


Fig. S6. Sensitivity analysis of the association between per 10-point increment CVH score and the risk of cancer with multiple imputation in men (A) and women (B). Models were fully adjusted for age, ethnicity, education level, annual household income, Townsend deprivation index, alcohol status, and baseline CVD status. Additionally, for women, the model was adjusted for menopausal status. The results were obtained from the integration of five sets of imputations: a, *P* <0.05; b, *P* <0.01; and c, *P* <0.001. CVH, cardiovascular health; CVD, cardiovascular disease.

Reference

1. Petermann-Rocha F, Ho FK, Foster H, et al. Nonlinear Associations Between Cumulative Dietary Risk Factors and Cardiovascular Diseases, Cancer, and All-Cause Mortality: A Prospective Cohort Study From UK Biobank. *Mayo Clin Proc*. 2021;96(9):2418-2431.
2. Wang X, Ma H, Li X, et al. Association of Cardiovascular Health With Life Expectancy Free of Cardiovascular Disease, Diabetes, Cancer, and Dementia in UK Adults [published correction appears in JAMA Intern Med. 2023 Apr 1;183(4):394] [published correction appears in JAMA Intern Med. 2023 May 15;:]. *JAMA Intern Med*. 2023;183(4):340-349.
